# In Search of Ideal Solutions for Cancer Diagnosis: From Conventional Methods to Protein Biomarkers in Liquid Biopsy

**DOI:** 10.3390/proteomes13040047

**Published:** 2025-09-23

**Authors:** Anca-Narcisa Neagu, Pathea S. Bruno, Claudiu-Laurentiu Josan, Natalie Waterman, Hailey Morrissiey, Victor T. Njoku, Costel C. Darie

**Affiliations:** 1 Laboratory of Animal Histology, Faculty of Biology, “Alexandru Ioan Cuza” University of Iași, Carol I bvd. 20A, 700505 Iasi, Romania; claudiujosan22@gmail.com; 2 Biochemistry & Proteomics Laboratories, Department of Chemistry and Biochemistry, Clarkson University, Potsdam, NY 13699-5810, USA; brunop@clarkson.edu (P.S.B.); watermnd@clarkson.edu (N.W.); morrisha@clarkson.edu (H.M.); njokuvt@clarkson.edu (V.T.N.); cdarie@clarkson.edu (C.C.D.)

**Keywords:** cancer detection, liquid biopsy (LB), protein biomarkers, proteomics

## Abstract

Cancer detection has made significant progress, moving from conventional methods to innovative, non-invasive or minimally invasive approaches aimed at improving early diagnosis, precision, and treatment outcomes. This review examines current and emerging diagnostic technologies, including liquid biopsy (LB), molecular biomarkers, and artificial intelligence (AI). LB analyzes biomarkers in bodily fluids, showing promise in detecting tumors at molecular levels, monitoring cancer progression, and predicting treatment responses. The assignment of specific proteoforms, often linked to tumor subtype, stage, and therapy resistance, adds another layer of diagnostic precision, offering valuable insights for personalized oncology. However, the clinical application of LB faces challenges related to sensitivity, specificity, tumor heterogeneity, and a lack of standardized protocols. Relatively high costs, complex result interpretation, and privacy concerns also hinder its widespread adoption in clinical practice. Despite these challenges, advancements in AI, nanotechnology, and multi-omics strategies offer opportunities to enhance cancer diagnostic accuracy. Future developments, including wearable biosensors and lab-on-a-chip technologies, could lead to personalized, real-time cancer detection with improved patient outcomes, potentially redefining cancer care and fostering a more proactive, patient-centered healthcare approach.

## 1. Introduction

### 1.1. Challenges of Conventional Cancer Diagnostic

Despite advances in cancer diagnosis, the search for a perfect or ideal method for the detection of malignant tumors remains still elusive. As a result, researchers and clinicians, along with patients actively involved in their care through participatory medicine [1], continue to seek more reliable tools, methods, and solutions. High-quality cancer screening is now focused on the early detection and treatment of precursor lesions, which are essential to reduce morbidity and mortality of cancer [2]. Through earlier cancer detection and with less discomfort, patients can be diagnosed and start treatment sooner, increasing the likelihood of successful outcomes and reducing both the psychological and physical impact of invasive procedures. 

Screening and diagnostic tests and/or methods in oncology are diverse and vary depending on the cancer type, stage, and healthcare systems. These methods include conventional imaging techniques (such as X-ray, ultrasound/ultrasonography (US), computed tomography (CT) and magnetic resonance imaging (MRI)), serum tumor biomarker analysis, and the histopathological analysis of tumor tissues. However, these methods still have several limitations, particularly related to sensitivity and specificity [3]. 

Despite significant advancements in cancer detection, management, and treatment monitoring, one of the major challenges remains the accuracy of screening and diagnostic methods. The effectiveness of these methods depends primarily on their sensitivity and specificity [4,5]. However, screening and diagnostic assessments are prone to errors, and misinterpretation by researchers and clinicians and can lead to serious consequences for both patients and healthcare systems worldwide [6]. In this context, it is crucial to minimize both false-negative and false-positive results, which can lead to overdiagnosis and overtreatment [2].

Sensitivity and specificity are fundamental metrics for assessing the accuracy of a diagnostic test [7]. The term sensitivity (true-positive rate) refers to the probability that a test will correctly identify a positive result when the patient has a certain disease, while specificity measures the probability that the test will correctly identify a negative result when the individual does not have the given disease [8]. Diagnostic accuracy evaluates how effectively a test distinguish between patients with and without the disease [7]. 

Lipscomb et al. (2022) proposed that an ideal test for screening large populations for multiple types of cancer must achieve clinically optimal sensitivity while minimizing false-positives [9]. For ovarian cancer (OC), Englisz et al. (2024) suggested that an ideal screening test should have a sensitivity greater than 75% and a specificity of at least 99.6% [10]. The reference standard, or gold standard, refers to the best available method or experimental model for reliable identifying a disease, against which the test index results are compared [7]. 

All methods used in cancer screening and diagnosis have inherent limitations. Conventional approaches are often suboptimal, emphasizing reduced sensitivity, specificity, and accuracy. They can be invasive, limited to certain cancer types, typically detecting one or a few cancer biomarkers. Additionally, these methods are not able to monitor dynamic changes in tumor development and fail to capture the clonal and molecular diversity within tumors. Invasive procedures also carry risks such as infections, bleeding, pain, and other complications related to anesthesia or recovery. Furthermore, they are time-consuming, labor-intense, and resource-consuming, with limited accessibility and high costs. These factors can result in late cancer detection, overdiagnosis, overtreatment, including unnecessary surgery, and delayed treatment decisions. Moreover, interpretation variability and human error can further compromise results.

### 1.2. Evolution of Cancer Diagnostic Technologies

Although the methods previously discussed remain fundamental in current oncological practice, cancer research continues to explore more precise, non-invasive or minimally invasive, and accessible tools for early and accurate diagnosis. To address these limitations, modern diagnostic methods are increasing being introduced. Cancer diagnostic has evolve from imaging-based techniques, including microscopic detection of malignant lesions, to the sophisticated analysis of tumor biomarkers in vitro and in vivo, using advanced mass spectrometry (MS) techniques, nano-devices, and innovative imaging technologies [11]. Proteoforms are increasingly recognized for their critical role in cancer biology [12]. They provide a more detailed molecular fingerprint of tumors than traditional biomarkers, reflecting the complexity and heterogeneity of cancer at different stages. Detecting proteoforms, particularly through non-invasive liquid biopsy approaches, holds great promise for improving early cancer diagnosis and enabling more precise patient stratification. As technologies for proteoform analysis continue to evolve, they offer valuable opportunities to enhance cancer detection and support the development of personalized treatment strategies. This review examines the evolution of cancer detection methods, from conventional approaches to emerging innovation in liquid biopsy (LB) and molecular biomarkers, with a focus on improving accuracy, minimizing invasiveness, and enabling early detection and personalized treatment.

### 1.3. The Promise of Liquid Biopsy and Future Directions

LB represents a technique that is transforming the way in which carcinogenesis and cancer development are detected and monitored. LB enables the accurate assignment of tumor-derived biomarkers, offering a less invasive alternative to conventional tissue/surgical biopsies [13]. The term ”liquid biopsy” is defined as a minimally invasive or non-invasive technique, focusing on blood/serum/plasma, urine, saliva, human breast milk, nipple aspirate fluid (NAF), tears, and other body secretions, to analyze tumor-derived molecules circulating in bodily fluids, including protein/peptides, proteoforms, autoantibodies, microRNAs (miRNAs), circulating tumor DNA (ctDNA), metabolites, as well as extracellular vesicles (EVs) and circulating tumor cells (CTCs), which can serve as biomarkers of cancer presence and progression [14]. Furthermore, the sensitive detection of CTCs through advanced platforms like magnetic beads, microfluidic chips or size-sensitive ultrafiltration is gaining attention as an effective biomarker-mediated tool for both cancer diagnosis and prognosis [15]. In particular, early-stage multi-cancer detection, such as for pancreatic, ovarian, and bladder cancers, has shown remarkable potential with a blood test based on EVs proteins, achieving an impressive 71.2% sensitivity and 99.5% specificity [16].

Compared to conventional tissue biopsies, LB has several advantages, including its ability to provide insights into the intratumoral heterogeneity because tumor-derived molecules are shed into bodily fluids from both primary and metastatic sites [17]. Thus, LB offers a comprehensive snapshot of the molecular landscape of cancer, reflecting the genetic and phenotypic diversity of tumors. One of the key advantages of LB is its high sensitivity, reproducibility, and flexibility, making it an attractive option for cancer detection and monitoring [18]. LB allows for repeated testing, enabling real-time monitoring of tumor dynamics, which is particularly valuable for continuous assessment of treatment efficacy, detecting minimal residual disease, and identifying potential relapses in patients.

Proteoforms, different molecular variants of proteins arising from genetic variations, alternative splicing, and posttranslational modifications (PTMs), identified through LBs provide important molecular information that can enhance early cancer detection and treatment [19]. Autoantibodies targeting these altered proteoforms serve as valuable biomarkers, reflecting the body’s immune response to cancer and offering potential for improved diagnosis and novel therapies [20]. A more detailed discussion of proteoforms and their roles is provided in a separate chapter.

LBs contain stable exosomal microRNAs (miRNAs), which represent a promising class of minimally invasive biomarkers for cancer diagnosis and prognosis [21]. These molecules offer valuable insights into disease progression and treatment response across multiple malignancies, including lung cancer, breast cancer (BC), prostate cancer (PCa), oral squamous cell carcinoma (OSCC), colorectal cancer (CRC), and others [21]. However, exosome-based research still faces several challenges, such as the complex, multistep process required from serum collection to miRNA analysis. These include difficulties in isolating tumor-derived exosomes and the lack of standardized methodologies for exosome separation and characterization [21].

In conclusion, LB has emerged as a promising method for detection of several cancer types, particularly in early stages of the disease, where conventional imaging and biopsy methods may fall short. Notably, LB has shown great potential for detecting early-stage BC [22], bladder cancer [18], hepatocellular carcinoma (HCC) [23], pancreatic cancer (PC) [24], and many other malignancies. Thus, LB are a promising tool for cancer diagnosis, prognosis, and patients stratification for personalized therapy [23]. In addition to its diagnostic applications, LB offers unique advantages in identifying therapeutic targets, analyzing drug resistance mechanisms, and monitoring multidrug resistance in advanced cancer patients [25]. Proteome-based plasma tests, for instance, have demonstrated higher accuracy when compared to other technologies and may serve as starting point for developing a new generation of screening tests capable of detecting multiple cancers at an early-stage within general population [26].

## 2. Limitations of Conventional Diagnostic Paradigms

Despite significant advancements in oncology, conventional diagnostic methods, such as histopathology, imaging, and single-analyte blood tests, continue to face critical limitations, particularly in the early detection of cancer, monitoring minimal residual disease, and capturing tumor heterogeneity. These approaches often rely on invasive biopsies, provide static snapshots of disease, and lack the sensitivity to detect low-abundance biomarkers or early molecular changes. Moreover, they are limited in their ability to guide personalized treatment decisions or adapt to dynamic changes in tumor biology. These shortcomings have underscored an urgent need for more sensitive, specific, and non-invasive diagnostic tools, prompting the development of innovative approaches such as liquid biopsies, multi-omics analyses, AI-assisted diagnostics, and point-of-care (PoC) technologies. These emerging methods aim to overcome the constraints of traditional diagnostics by offering real-time, comprehensive, and minimally invasive insights into tumor biology, thus paving the way for earlier detection, better monitoring, and more precise cancer management. These limitations are discussed in detail in this chapter, highlighting how they have driven the development of more advanced and innovative diagnostic strategies.

### 2.1. Serum Biomarkers Limitations

Serum and plasma are highly complex mixtures containing thousands of proteins, with some being abundant and others present in much lower concentrations, making detection challenging. Mass spectrometry (MS) play a crucial role in the assignment of cancer biomarkers in human serum due to its ability to detect a wide range of proteins and peptides, including variants [27]. Circulating serum markers, produced by both normal and abnormal cells or tissues, are often considered attractive for clinical use as they can be obtained through minimally invasive methods [23]. While several serum markers have been validated for diagnosis and monitoring cancer progression, none exhibit the necessary specificity, sensitivity, and predictive value required for population screening [27]. 

For instance, prostate-specific antigen (PSA), alpha-fetoprotein (AFP), cancer antigens such as CA-125, CA-15.3, CA-19-9, and carcinoembryonic antigen (CEA) are still commonly used for detecting prostate, liver, ovarian, breast, pancreas, or colon cancers, respectively, despite their limitation in accuracy and early-stage detection [27].

PSA is sensitive but poorly specific for PCa detection in symptomatic patients, often leading to overdiagnosis and treatment of latent cancer without clinical evidence. This is due to its inability to distinguish between indolent and aggressive forms of PCa [28,29]. Serum PSA is an example of a widely used biomarker for screening, diagnosis, and prognosis of PCa. However, it is considered an organ-specific biomarker, even if a study of nipple aspirate fluid (NAF) found that women with no risk factors of BC had high levels of PSA, while women with precancerous or invasive lesions had reduced PSA levels [30]. PSA is non-cancer specific and its levels can also be elevated in non-cancerous conditions, such as urinary tract infection, prostatitis and benign prostatic hyperplasia (BPH) [31,32].

Furthermore, serum PSA can be influenced by a variety of internal and external factors. To address these limitations, several fluid-based molecular biomarkers have been developed for PCa detection [33]. One such biomarker is the Prostate Health Index (PHI), which combines total PSA, free PSA, and [-2] proPSA (p2PSA) isoform into a score derived from a simple, inexpensive blood test [34]. PHI has been shown to improve detection of high-grade PCa compared to PSA-based assays alone [35]. Another example is the 4Kscore blood test, which integrates four kallikrein protein biomarkers (total PSA, free PSA, intact PSA, and human kallikrein-related peptidase 2) into an algorithm to enhance the prediction of aggressive form of PCa. This allows men with low 4Kscore results to safely delay biopsy [36]. The prostate cancer antigen 3 (PCA3), which is highly expressed in prostatic tumors, can be measured through a urine test. The PCA3 test offers acceptable sensitivity and specificity, making it a valuable non-invasive method for PCa diagnosis [37].

Alpha-fetoprotein (AFP) is a glycoprotein that is often elevated in the sera of patients with liver cancer [38]. However, elevated serum AFP have also been reported in patients with other cancers, including BC, esophageal, cervical, pancreatic, endometrial, gastric, lung, and rectal cancers [38]. Additionally, AFP can be elevated in non-cancerous conditions, such as cirrhosis, nephrotic syndrome, and gastritis [38]. Similarly, serum CA-125 is an extensively used biomarker for OC diagnosis. However, CA-125 levels may also be elevated in other malignant and non-cancerous conditions, such as endometrial, breast, pancreatic, and gastrointestinal cancers, as well as liver cirrhosis, endometriosis, and large uterine fibroids/leiomyomas [10,39,40]. Furthermore, CA-125 levels were found to be significantly higher in patients with polycystic ovarian syndrome (PCOS) compared to control patients, with a value of 11.45 U/L showing high sensitivity (96.7%) and specificity (87.1%) for PCOS [41]. This suggests that CA-125 could be used as an additional diagnostic biomarker for this disease [41]. Additionally, Kim et al. (2015) reported that higher plasma volumes in obese patients could dilute serum tumor biomarkers such as PSA for PCa, and CA-125 and CA-19-9, which can affect the efficiency of tumor screening for ovarian and pancreatic cancer [42]. Despite these challenges, serum CA125 has shown the highest sensitivity (93.30%), while human epididymis protein 4 (HE4) has demonstrated the highest specificity (97.87%) in diagnosis of epithelial ovarian cancer (EOC) [43]. Additionally, CA-125 and HE4 are clinically approved biomarkers for the diagnosis and prognosis of endometrial cancer (EC) [44]. In these cases, the combined detection of serum soluble epidermal growth factor receptor (sEGFR), CA-125, and HE4 has been shown to increase the specificity and efficiency in EOC diagnosis [43].

Carcinoembryonic antigen (CEA) was originally identified as a colon cancer antigen, and elevated serum CEA levels have led to its clinical use as a diagnostic biomarker for CRC [45]. However, CEA levels are also elevated in cancer such as pancreatic and rectal cancers, as well as in non-cancerous conditions like lung fibrosis, uremia, chronic obstructive pulmonary disease (COPD), and Alzheimer’s disease [45]. Furthermore, CEA levels are known to increase with aging [45].

For early cancer detection, risk assessment, and therapy-related monitoring, improving serological biomarkers across different cancer types is essential [46]. LB offers significant advantages over conventional serum biomarkers due to its ability to capture a broader spectrum of molecular information, including mutations and PTMs of proteins that may be overlooked by conventional serum markers. As a result, LB can provide a more comprehensive understanding of tumor mechanisms and support the development of personalized treatment strategies. Therefore, advances in innovative proteomics technologies are urgently needed to facilitate biomarker discovery in tumor-associated LBs [47].

High-plex proteomics technologies, ranging from MS and antibody/antigen arrays to innovative approaches like aptamer-mediated LB [48], proximity extension assay (PEA), and reverse phase protein arrays (RPPA), are capable of simultaneously measuring hundreds of proteins in LB samples and can detect cancer at early stage [47]. These technologies exploit highly accurate and unbiased affinity-based biomolecule capture of from LB, primarily relying on nucleic acid aptamers, synthetic DNA or RNA oligonucleotides with unique tertiary structures that selectively target proteins and other molecules. Aptamers leverage the specificity and binding affinity of ligands toward surface receptors on circulating targets [48,49]. For detecting novel cancer biomarkers, affinity-based methods enable large-scale proteome analysis in complex bodily fluids, overcoming the limitations of conventional, and even advanced, MS-based proteomics [46]. Roy et al. (2021) and other authors demonstrated that nucleic acid aptamers exhibit high specificity and affinity, as well as modifiability and flexibility, making them easily adaptable for evolution and functionalization. These characteristics make aptamers a reliable alternative to antibodies for diagnostics, imaging, and targeting applications, positioning them as ideal recognition elements for LB [48,50]. Monoclonal antibody production is more expensive than the process of fabricating and improving aptamers, which can be successfully used for immunohistochemistry and LB [51]. Consequently, aptamer-based recognition of BC cells opens a new era for BC diagnosis [51]. High-multiplex aptamer-based serum proteomics can accurately discriminate between patients with OSCC and healthy controls, achieving 100% accuracy. This approach demonstrates that panels of OSCC-associated proteins revert to levels seen in healthy controls after tumor resection, identifying candidate cancer-specific serum biomarkers [46]. Moreover, aptamer-based microfluidic platforms or chips for detecting CTCs and EVs hold great potential as powerful tools in clinical applications [52]. Aptamers have emerged as cost-effective detection tools for targeting HE4, which is overexpressed in OC, making them a promising strategy for OC diagnostics or the development of urine biosensor-based tests for OC [53]. Additionally, the development of cancer-specific smart aptamers enables the targeted delivery of therapeutic molecules directly to tumor cells, minimizing damage to healthy cells and tissues [54,55].

In conclusion, despite significant progress in the discovery and validation of serum biomarkers for cancer, traditional approaches based on canonical proteins remain limited by issues of specificity, sensitivity, and clinical utility. As shown with markers such as PSA, AFP, CA-125, and CEA, many of these proteins are elevated not only in malignancies but also in a variety of benign or unrelated conditions, leading to overdiagnosis, false positives, and poor predictive value, particularly in early-stage disease. These limitations stem from a gene-centric view of the proteome, where proteins are treated as direct correlates of gene expression, ignoring the functional diversity generated by alternative splicing, PTMs, and proteolytic processing. In reality, proteomes are composed of proteoforms, distinct molecular species of proteins that represent the actual biological effectors within cells and tissues. Therefore, effective biomarker discovery must move beyond protein-level assignement toward proteoform-level resolution, where these functional differences can be captured and quantified [56].

Conventional MS-based approaches, especially bottom-up methods, often fall short in capturing this complexity. Bottom-up (BUP) MS enables high-throughput protein assignment but lacks the resolution needed to distinguish proteoforms, isoforms, and complex PTMs due to peptide-level limitations [57]. However, integrative top-down proteomics (iTDP) has emerged as the only analytical strategy currently capable of routinely detecting and quantifying the broad diversity of proteoforms in native biological samples [58]. While iTDP offers the necessary depth and resolution, it comes with trade-offs, such as higher complexity, cost, and reduced throughput compared to faster, lower-resolution methods.

Biomarker discovery stands at a crossroads: prioritizing speed and scalability often comes at the cost of accuracy and biological relevance, while rigorous, high-resolution analyses better capture the complexity of the proteome. Although slower, proteoform-level data provide deeper insights and more selective biomarkers. High-plex technologies like aptamer arrays and PEA expand coverage but must maintain high analytical standards [59]. Embracing proteome complexity is essential for advancing precision oncology in the post-proteogenomic era [56].

### 2.2. Histopathology Limitations

Histology, including histopathology, immunohistochemistry (IHC), electron microscopy (EM), and immunocytochemistry (ICC), plays a crucial role in clinical diagnosis in oncology. However, there are cases where histology alone cannot provide a definitive diagnosis, requiring multidisciplinary approaches that involve expert teams for accurate results [60,61]. To enhance tumor detection and management, molecular biomarkers and genetic analysis are essential in complementing histopathological findings with molecular diagnostics [61].

Histological analysis typically requires a biopsy to obtain a tumor tissue sample, a procedure that can be invasive and associated with complications such as infections, bleeding, or pain. This is particularly problematic for patients undergoing invasive biopsy procedures, such as core needle biopsies or surgical biopsies, which necessitate longer recovery times. Additionally, biopsy may not be feasible or accessible in certain cases, such as with deep-seated tumors, a high risk of bleeding, or multiple tumor sites [62]. Conventional histopathological tissue diagnosis methods are also time-consuming and labor-intensive, often taking several days, which can delay both diagnosis and treatment decisions [63].

This delay can be particularly problematic in urgent cases where rapid decision-making is crucial. Additionally, a biopsy samples only a small portion of the tumor tissue, which may not be representative to the entire tumor, especially given that tumors are often characterized by high genetic and molecular intraheterogeneity. In such cases, a biopsy may fail to capture the full molecular diversity present within tumor, potentially compromising the accuracy of the histopathological diagnosis. Moreover, early lesions or small tumors may be missed, affecting the detection of early-stage cancer or micrometastases.

Histological analysis typically provides a snapshot of the tissue at the time of the biopsy but does not offer a prospective outlook on tumor progression [64]. To monitor tumor changes over time or detect recurrence, multiple biopsies may be required, which can be invasive and, in some cases, impractical. Additionally, the use of 2D tissue sections can limit the ability to analyze the tumor in its three-dimensional context. However, recent advances in microscopic and computational technologies now enable successful 3D reconstruction of tissues and tumors, improving the scope of analysis [64]. In certain types of cancer, histology alone may not provide sufficient diagnostic or prognostic information.

Although histopathology is a crucial diagnostic tool, widely regarded as the ”gold standard” for cancer diagnosis, biopsy results can still be variable, often subjective, and semi-quantitative [65,66]. As a result, a second opinion from an additional pathologist is often sought [66]. Tseng et al. (2023) found that seeking a second opinion can lead to changes in the diagnosis, with shifts from benign to malignant diagnoses in 10–19% of cases [66]. Additionally, partial or minor disagreement regarding the grade or subtype of the tumor occurred in 20–34% of cases, while significant changes in prognosis or the treatment occurred in 17–39% of cases [66]. The same authors also highlighted that interpreting of histological margins of tumors, especially when tissue processing is inadequate, presents a challenge for pathologists. These interpretations can be time-consuming and, at times, subjective [66].

It is estimated that 85% to 95% of breast lesions biopsied show benign histology, and the costs associated with these procedures can be considerable [67]. For BC detection, fine needle aspiration cytological biopsy (FNAC/FNAB) and core needle biopsy (CNB) are commonly used prior to surgical biopsy and are generally considered relatively inexpensive diagnostic procedures [68]. Other minimally invasive sampling methods have become more preferred, including ultrasound guided FNAB (US-FNAB) and image guided CNB (IG-CNB) [67]. A systematic review and meta-analysis by Wang et al. (2017) evaluating suspicious breast lesions concluded that CNB has better sensitivity (95%) compared to FNAC (74%), although the specificity of CNB is similar to FNAC (98% vs. 96%) [69]. Silva et al. (2023) reported that the sensitivity, specificity, and accuracy of FNAC (97%, 94%, and 95%, respectively) were comparable to CNB (97%, 96%, 96%, respectively) as CNB, with FNAC showing lower complication rates [68]. A comparative high-throughput proteomic analysis of matched CNB, FNAC, and surgical resection tissue samples from BC patients revealed that about 85% of proteins were identified in all sample types, indicating that FNAC is suitable for proteomic analysis and holds promise as a source for assignment new protein biomarkers for BC [70]. Several proteins, including cyclin D1 (CCND1), cyclin-dependent kinase (CDK6), HER2, and insulin-like growth factor 1 receptor (IGF1R), were found in higher quantities in FNAC compared to tissue samples, while tubulin beta 2A (TUBB2A) was detected exclusively in FNAC [70]. 

Immunohistochemistry (IHC) is an essential method for detecting specific diagnostic proteins within tissue samples, but it remains limited to detecting one biomarker per tissue section or, in dual-color IHC assays, two biomarkers simultaneously [71,72,73]. While multiplexed immunofluorescence (IF) can visualize multiple proteins at once, it is constrained by spectral overlap. These challenges can be addressed using Fluorescence Lifetime Multiplexing (FLEX), which allows for the differentiation of 11 or more biomarkers in 3D tissues [71]. Despite being a qualitative and subjective approach, IHC continues to be a powerful tool for detecting the in situ distribution of biomarkers in FFPE tissue sections. However, IHC also has several limitations, including a lack of standardized guidelines for staining, which leads to variability in results across different laboratories due to the use of different antibodies or protocols [74]. 

For instance, the accurate and reliable assessment of human epidermal growth factor receptor type 2 (HER2) status, along with other hormone receptors (HRs) such as estrogen receptor (ER) and progesterone receptor (PR), is crucial for stratifying cancer subtypes and determining appropriate treatments. However, the inter-assay accordance for detecting HER2-low tumors is not high. Even with the same staining assay, various factors can influence the result of HER2 IHC-based detection [75,76]. 

Consequently, it is critical to accurately identify HER2-low cancer in HER2-negative patients [76]. Studies have shown that factors such as the type of fixative used, as well as the time to and duration of fixation of tumor tissues can impact HER2 staining intensities and FISH scores [75,76]. Additionally, changes in antigen retrieval and antibody incubation procedures can affect staining intensity of HER2 expression [76]. A significant discordance in HR positivity rates has been reported between IHC analysis (84%) and enzyme immunoassay (EIA) (45%) in invasive lobular carcinoma, concluding that IHC is more sensitive, specific, and cost-effective than EIA [77]. Accurate evaluation of HER2 status is essential for patient stratification and precision therapy, and IHC remains one of the most commonly used pathological method for HER2 status [65]. In this context, Thanasan et al. (2023) recommended using IHC alone for patients with BC undergoing anti-HER2 treatment, even without in situ hybridization (ISH), due to the strong concordance between HER2 IHC scores and HER2 amplification, with a sensitivity of 87.96%, specificity of 93.75%, and an accuracy of 89.74% for BC diagnosis [78]. Furthermore, a meta-analysis by Shamshirian et al. (2020) revealed serum HER2 levels have low sensitivity (53.05%) but reasonable specificity (79.27%) and an accuracy of 72.06% for BC diagnosis. This suggests that assessment of serum HER2 levels can serve as a valuable verification test [79]. 

Molecular characterization is crucial for a better understanding of tumor characteristics [80]. Mass spectrometry imaging (MSI) is as an excellent method for the accurate detection and mapping of tissue biomarkers in oncological applications [80], and it is a powerful tool for investigating tumor aggressiveness based on cancer biomarkers [81]. Additionally, MSI allows for the analysis of histopathological features without the need of specific antibodies [80]. Using MSI-based techniques, Gonçalves et al. (2023) demonstrated that vimentin (VIM) overexpression is associated with triple-negative breast cancer (TNBC), a finding validated by IHC, suggesting that VIM could be a promising target for BC diagnosis and treatment [80]. 

These histology-related limitations underscore the need for complementary technologies, such as LB or advanced imaging techniques, to enhance cancer screening, detection, and management. The main advantages of LB vs. histopathology are listed in Table 1. 

### 2.3. Imaging Methods Limitations

The majority of breast tumors are initially detected by patients through breast self-examination (BSE). Although recommendations suggest that women should continue performing BSE, even if its efficacy remains controversial, as research has not clearly linked BSE with significant benefits in early detection [82]. Studies have shown that BSE has low estimated sensitivity (20% to 30%), and its effectiveness tends to decrease among older women [83]. BSE remains one of the first steps women take in detecting abnormalities of the breast.

While clinical breast examination (CBE) performed by an experienced health examiner is the next option, it also has limitations in sensitivity. An alternative is the Intelligent Breast Exam (iBreastExam or iBE), which electronically palpates the breast to identify possible abnormalities. This device has been shown to have significantly better sensitivity than CBE (63% vs. 31%) [84]. Recent studies, such as by Bhimani et al. (2023), demonstrated that iBE, a portable, wireless, radiation-free, cost-effective, easy-to-use, and hand-held device, could be a valuable tool for BC detection. Sensitivity with iBE ranges from 34.3% to 86%, and specificity ranges from 59% to 94% [85]. This device has moderate to higher diagnostic ability for malignant lesions, with a sensitivity for identifying breast tumors ranging between 57% and 93%, allowing for the identification of breast tumors ranging between 0.5 cm to 9 cm [85].

Imaging plays a critical role in BC diagnosis, with mammography being the gold standard. However, ultrasonography (US) is increasingly emerging as a routine alternative or complementary imaging method [86]. Sood et al. (2019) demonstrated that US can be an effective primary detection tool for BC, especially in low-resources settings or where cost is a consideration. The overall sensitivity and specificity of US were reports to be 80.1% and 88.4%, respectively [87]. US is particularly beneficial for BC diagnosis in women with dense breast tissue, where other imaging methods may be less effective [87]. 

When it comes to imaging methods for BC screening, X-rays are used in both film mammography and digital mammography [88]. However, film mammography has limited sensitivity in detecting BC, particularly in women with dense breast. In these cases, digital mammography offers significant improved accuracy [89]. For instance, in a study involving Japanese women aged 40–49, the sensitivity of modern film mammography alone was found to be 44.1% for women with dense breasts and 34.8% for women with non-dense breasts [90]. Digital mammography has a sensitivity of 97%, specificity of 64.5%, and diagnostic accuracy of 89.3%, making it an accurate tool for detecting BC. However, mammography is not always feasible or available, particularly in certain regions or settings [87,88]. In 2015, in the Journal of the Royal Society of Medicine, Gøtzsche argued that mammography screening could be more harmful than beneficial. The author suggested that it does not increase the women’s lifespan, instead contributing to overdiagnosis and leading to unnecessary mastectomies [91]. According to Gøtzsche, if mammography-screening were a pharmaceutical drug, it would likely have been withdrawn from the market due to its negative effects [91].

Computed tomography (CT) is a widely used non-invasive imaging modality that plays a crucial role in the diagnosis of various diseases, including cancer [92]. Despite the fact that CT scans expose patients to ionizing radiation, which can potentially lead to carcinogenic mutations and DNA damage, they remain an invaluable tool in cancer diagnosis worldwide [92,93]. A meta-analysis involving 111.6 million adult participants from Europe, America, and Asia found a dramatically increase in cancer risk associated with CT scans, with the risk being positively correlated with the radiation dose and the CT sites [93]. The long-term effects of radiation exposure can manifest over several decades [92]. However, some studies indicate that exposure to multiple CT scans and other diagnostic radiologic imaging (10–20 scans) with a cumulative dose of 100-200 mSv does not significantly increase cancer risk [94].

A recent study evaluating 144 cases of CT scans correlated with histopathological findings showed a sensitivity of 98.52% and specificity of 87.50% for CT scans. The precision and accuracy of CT scans were reported as 99.25% and 97.91%, respectively [95]. In the context of lung cancer screening, low-dose CT demonstrated a sensitivity of 88.9% and specificity of 92.6%, while chest X-ray had a sensitivity of 78.3% and specificity of 97.0% [96]. These findings indicate that low-dose CT screening for lung cancer has higher sensitivity but slightly lower specificity compared to conventional chest X-rays [96]. In another study analyzing 142 pediatric whole-body MRI results, the sensitivity for detecting malignancies was 93.8%, with a specificity of 93.4%. The positive predicted values was 65.2%, while the negative predicted value was 99.1% [97]. In BC diagnosis, Aziz et al. (2022) examined the concordance between radiological and histopathological findings. They reported a sensitivity of 94.48% and specificity of 43.14% for Breast-Imaging and Reporting Data System (BI-RADS) in relation to mammography and breast US, highlighting the significant correlation between radiological and histopathological results in invasive BC [98].

F-18 fluorodeoxyglucose (FDG) PET/CT has been shown to have superior diagnostic performance compared to conventional imaging methods for detecting distant metastases in BC. Specifically, FDG PET/CT exhibits a higher sensitivity (97.4%) and specificity (91.2%) compared to conventional imaging, which has sensitivity and specificity of 85.9% and 67.3%, respectively [99]. In the context of detection of head and neck squamous cell carcinoma (HNSCC), the latest-generation FDG-PET/CT demonstrates improved sensitivity in detecting cervical lymph node (LN) metastasis, while specificity remains similar or slightly lower than older PET/CT scanners [100].

Prostate-specific membrane antigen (PSMA) is a type II transmembrane glycoprotein typically expressed in the cytoplasm of normal prostate cells. It is overexpressed on almost all PCa cell membranes, and this overexpression is correlated with increased DNA repair mutations and the development of hormone-resistant PCa [101]. PSMA PET/CT, a next-generation imaging modality, has significantly improved the clinical diagnosis of PCa across various clinical settings. It offers greater sensitivity and specificity compared to conventional imaging methods such as CT, MRI, and bone scintigraphy [102,103].

However, one of the challenges with PSMA PET/CT is limited number of specialists trained to interpret these scans, and there is variability in data interpretation among different readers [104]. To address these challenges, AI-based techniques can be employed to assist in the interpretation of PCa imaging. These AI methods can aid in detecting primary tumors, interpretation, including detection of primary tumor, identifying local recurrences, assessing metastatic lesions, classifying lesions, and even predicting or prognosticating disease outcomes [104].

LB has emerged as a crucial tool in the management of PCa, using prostatic secretions, urine, and blood as sources for biomarker discovery, validation and clinical implementation [33,105]. Numerous urinary biomarkers have been proposed for PCa detection, enhancing diagnostic accuracy and patient monitoring [106]. A study by Höti et al. (2023) demonstrated that urinary PSA, when combined with serum PSA, exhibited a higher predictive ability in differentiating PCa from other conditions [107]. The authors found that patients with aggressive PCa had lower levels of urinary PSA compared to those with non-aggressive cancer. In contrast, serum PSA levels were significantly higher in patients with aggressive cancer [107]. Additionally, the study revealed that aggressive PCa was negatively correlated with PSA expression in PCa tissues as analyzed by IHC [107].

In the current era of proteomics, mass spectrometry (MS)-based technologies, such as matrix-assisted laser desorption/ionization MS (MALDI-MS), surface-enhanced laser desorption/ionization MS (SELDI-MS), and multiplexed isobaric tagging technology for relative quantitation (iTRAQ), enable for detection and quantitative analysis of the PCa proteome. These technologies are shifting the focus from a single biomarker (typically PSA) to panels of protein biomarkers, which necessitate the discovery of novel biomarkers for improved diagnosis and patient stratification [108]. It is well known that a single biomarker, while providing its own sensitivity and specificity, is insufficient for accurately diagnosing and stratifying PCa [29]. Consequently, the use of multiple biomarkers can enhance diagnostic accuracy compared to individual biomarkers [29].

In a study by Franzi et al. (2023), capillary electrophoresis coupled with mass spectrometry (CE-MS) profiling was used to develop a multi-dimensional biomarker model that included 181 urinary peptide biomarkers, which demonstrated strong accuracy in detecting PCa [106]. Many of these peptides were derived from collagen proteins, indicating the involvement of extracellular matrix in tumor invasion and progression. Other biomarkers included proteolytic products of proteins such as S100A9, uromodulin, albumin, fibrinogen alpha, mucin-2, beta-2-syntrophin, alpha-1-acid glycoprotein 1, protein phosphatase 1 regulatory subunit 3A, while markers like gelsolin, insulin-like growth factor II, and prostaglandin-h2-isomerase were found to be decreased in the urine of PCa patients compared with non-PCa group [106].

Additionally, using liquid chromatography coupled with mass spectrometry (LC-MS) to profile endogenous peptides in urine samples from patients with PCa, benign prostate hyperplasia (BPH), and healthy individuals, De Souza Dutra et al. (2023) identified five urinary peptides derived from uromodulin that are less abundant in the urine of PCa patients and might be considered as putative PCa biomarkers, able to differentiate between PCa and BPH [29]. These urinary peptides were shown to have high sensitivity (81.82%) and specificity (88%), outerperforming PSA in distinguishing between PCa and BPH [29]. The main advantages of LB vs. computed tomography (CT) are listed in Table 2.

## 3. What’s Innovative in Cancer Detection and Management?

**Figure 1:**
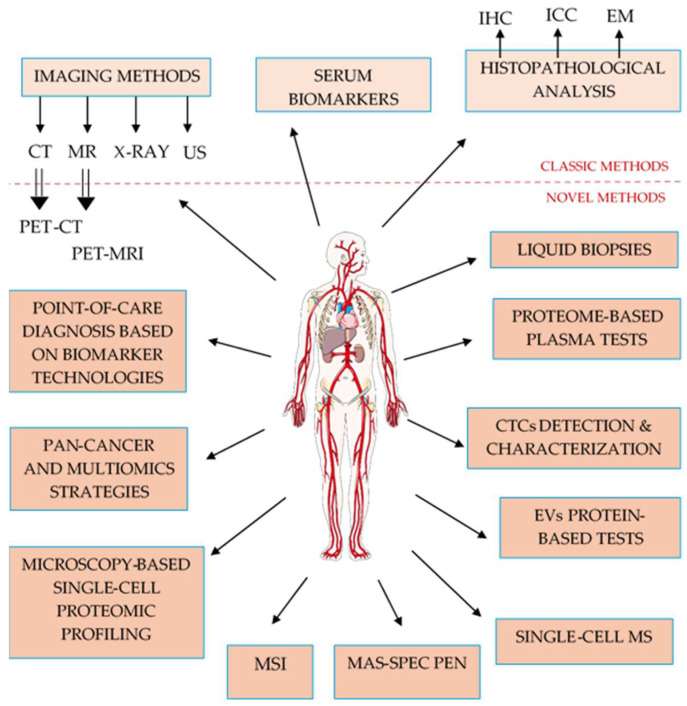
Conventional vs. modern methods used in cancer detection and management. CT—computed tomography; CTCs—circulating tumor cells; EM—electron microscopy; EVs—extracellular vesicles; ICC—immunocytochemistry; IHC—immunohistochemistry; MRI—magnetic resonance imaging; MS—mass spectrometry; MSI—mass spectrometry imaging; PET-CT—positron emission tomography-computed tomography; PET-MRI—positron emission tomography-magnetic resonance imaging; US—ultrasonography; X-ray—radiography.

Innovation in cancer detection and management lies in the integration of advanced technologies and methodologies that enhance early diagnosis, improve accuracy, and minimize invasiveness. These alternatives to conventional methods enable the non-invasive detection of cancer, offering a real-time snapshot of tumor genome, transcriptome, proteome, and metabolome. They also enable early identification of cancer even before clinical symptoms appear. Advanced imaging technologies offer greater precision in detection of malignancy at the molecular level, improving early diagnosis and correct staging. Artificial intelligence (AI) and machine learning (ML) are increasingly integrated into diagnostic platforms, analyzing vast amounts of medical data, which expand rapidly due to advances in digital health tools and analytics. In this context, the use of nanotechnologies has emerged as a complementary innovation, offering new possibilities for improving diagnosis, treatment, and monitoring in oncology. Together, these innovative approaches (Figure 1) will be reviewed in the following subchapters, highlighting their significant potential to transform the landscape of cancer detection, and lead to better treatment decisions and personalized care for each patient.

### 3.1. Established Clinical Standards as a Foundation for Innovation

While established clinical standards, such as multimodal imaging (e.g., CT, MRI, PET), targeted molecular assays, and histopathology remain the cornerstone of cancer diagnosis and management due to their validated clinical utility, they are increasingly being augmented by innovative approaches. These novel methods aim not to replace, but to complement and enhance current diagnostic workflows, enabling earlier detection, better stratification, and more precise therapeutic interventions.

- Multimodal imaging

To enhance diagnostic accuracy across various cancer types, the combination of different imaging techniques has been revolutionary, providing a more precise and comprehensive view of tumors. The latest-generation ^18^F-fluoro-2-deoxy-glucose positron emission tomography/computed tomography (^18^F-FDG PET/CT) offers improved sensitivity and image quality compared to older PET/CT scanners [100]. Furthermore, due to its higher sensitivity, the ^18^F-FDG PET/CT scan plays a synergistic role with MRI in diagnosing sub-centimeter malignant lesions [109]. The combination of PET and MRI in PET/MRI hybrid technology offers distinct advantages over PET/CT, including the absence of ionizing radiation, enhanced soft-tissue contrast, a wide range of acquisition sequences, and potential superiority in specific body areas and specific situation, such as tumor recurrence detection [110]. For instance, whole-body ^18^F-FDG PET/CT imaging has limitations in detecting brain metastasis form non-central nervous system tumors due to high background activity, making MRI scanning a valuable complementary tool [111]. Emerging combinations with artificial intelligence (AI) or molecular probes are pushing multimodal imaging toward next-generation diagnostics, offering enhanced sensitivity and precision.

- Genetic and genomic testing

Genetic biomarkers are playing an increasingly important role in early cancer detection, prognosis assessment, and treatment planning or management, supporting the development of targeted therapies aimed to improve patient outcomes. Thus, genetic testing, which analyzes gene expression profiles and mutations, has become an important tool in improving the accuracy of cancer diagnosis and guiding personalized treatment strategies [112]. The decision between single/limited gene testing and multigene panels depends on clinical context [113]. For example, in early-stage BC, multigene assays (MGAs) are utilized to support decisions regarding adjuvant therapy and to help estimate patient prognosis, while for individuals with metastatic BC, testing for specific genetic alterations can identify therapies with potential efficacy [112]. For instance, multigene prognostic genomic assays have become essential in managing early-stage BC [114]. Notable examples include the Oncotype DX Recurrence Score^®^ (RS), also known as the 21-gene recurrence score assay, widely used in Europe and America [112,115], MammaPrint^®^ (MMP), also known as the 70-gene risk of distant recurrence signature [112,116], Prosigna^TM^, a PAM50-based subtype classifier and risk model [117], EndoPredict^®^, a 12-gene molecular score (MS) [118], and Breast Cancer Index (BCI), a gene-expression based assay that evaluates tumor proliferation pathways and estrogen signaling in BC [119]. The NanoString nCounter^®^ DX Analysis System offers a digital profile of up to 800 genes, providing more precise and accurate measurements of mRNA expression levels in formalin-fixed paraffin-embedded (FFPE) specimens compared to PCR-based methods [117]. Tissue-based gene expression tests also increasingly used to estimate PCa aggressiveness [120]. Recently, McHugh et al. (2025) published a study about the assessment of a Polygenic Risk Score in screening for PCa [121].

Thus, the advent of next-generation sequencing (NGS) technology allows multigene testing for hereditary cancer risk [113]. Current clinical cancer genome sequencing typically relies on targeted exome panels, which focus on protein-coding regions representing only a small fraction of the genome [122]. As a result, somatic mutations in non-coding regions—such as regulatory elements, non-coding RNAs, and mitochondrial DNA—remain largely unexplored [122]. With technological progress, emerging applications, such as fragmentomics, a new field studying fragments of circulating DNA (cirDNA) for cancer screening, are gaining attention [123]. Circulating tumor DNA (ctDNA) has emerged as a promising tool for cancer detection, prognosis, and treatment monitoring through liquid biopsy [124]. This approach captures tumor-derived DNA from both primary and metastatic sites and allows for the detection of genetic and epigenetic alterations [124]. Additionally, structural variants and viral integrations in cancer genomes are not yet comprehensively studied [122]. To solve these inconvenient, the appropriate use of genomic testing, such as whole-genome sequencing (WGS), whole-exome sequencing, or multigene tumor profiling, has become increasingly important in modern oncology [125]. Unlike traditional genetic testing, which primarily focuses on identifying germline mutations linked to cancer risk, genomic testing provides a comprehensive analysis of somatic alterations within the tumor itself [125]. This enables more precise diagnosis, prognosis, and selection of targeted therapies, making it a key tool in the era of personalized medicine.

WGS is known as a powerful NGS tool with large applications in cancer research field [126]. WGS enables a wide range of analyses, including detection of non-coding mutations (e.g., in promoters and regulatory regions), large structural variants (deletions, insertions, inversions, duplications, translocations), viral genome integration, mitochondrial alterations, and immune-related features like HLA typing [122]. It also supports the diagnosis and treatment of hereditary cancers through germline variant analysis, pharmacogenomics, and mutational signature profiling [122]. Whole genome and transcriptome sequencing (WGTS) provides superior diagnostic and therapeutic insights compared to gene panel testing by detecting a broader range of relevant genetic alterations [127]. It enables prediction of tissue-of-origin in most previously undiagnosed cases and informs treatment decisions for a higher proportion of patients. Additionally, WGTS applied to cell-free DNA shows promise as a feasible method for guiding clinical management [127].

The disadvantages of genetic and genomic testing include the ethnic disparities that may impact MGA testing outcomes [112]. Moreover, there are limitations to genomic testing, primarily related to their high costs, which can range from 2000 US$ to 4000 US$ [116,125]. Although ctDNA shows potential as a biomarker in various cancers, challenges remain due to its low concentration, short half-life, and the lack of standardized cut-off values [124]. A major challenge is expanding the use of NGS to all cancer patients to support informed treatment decisions [128]. Key concerns include tissue sample quality, preservation, and health technology assessment [128]. WGS also has limitations, such as shallow read depth—risking false negatives in highly heterogeneous tumors—the need for frozen tissue, large data volumes, high analysis and storage costs, and difficulties in interpreting novel variants [122]. These challenges are being addressed by long-read sequencing technologies, which improve structural variant detection, and deep learning methods like convolutional neural networks, which enhance variant interpretation [122]. 

Genetic test misinterpretation is common and can affect clinical decisions [129]. Challenges often involve unclear reports, limited counseling, and variants of unknown significance. Improved communication and further research are needed to reduce errors. In conclusion, genomic testing can improve cancer care by guiding risk assessment, prognosis, and therapy, yet its use remains limited due to difficulties in result interpretation and lack of clear guidance for non-geneticists [130].

- Liquid biopsy sources for detection of protein biomarkers

LB are increasingly used alongside traditional biopsies, offering the advantage of detecting disease progression months before it becomes visible through imaging techniques or clinical evaluation [21]. Detection of proteins and their diverse proteoforms, including autoantibodies, in LBs offers valuable insights into tumor biology, serving as sensitive and specific biomarkers for early cancer diagnosis, disease monitoring, and personalized treatment strategies. 

Over the past decades, significant efforts have been dedicated to exploring, discovering, and validating novel, sensitive, specific, and accurate protein biomarkers for cancer diagnosis, prognosis, and targeted therapy [131]. Investigating tumor protein biomarkers offers several advantages over conventional cancer detection techniques, such as serological markers, biopsy, and imaging. The combinatorial benefits of tumor protein biomarkers include their non-invasive or minimally invasive nature when assessed through body fluids, like LB, enabling early-stage cancer detection. Additionally, they provide high capacity for monitoring treatment responses, guiding personalized and targeted therapies, and supporting precision oncology. Protein biomarkers are also valuable for detecting minimal residual disease, reducing radiation exposure, enabling risk stratification, and improving quantification and prediction. Their broad applicability extends to rare cancers and tumors located in difficult-to-assess organs, offering a cost-effective and less discomforting alternative for cancer patients.

Proteoforms detected through LB provide critical, detailed molecular insights that enhance early cancer diagnosis, enable more precise disease monitoring, and support personalized treatment strategies. Autoantibodies targeting tumor-associated proteoforms represent a particularly valuable class of biomarkers, reflecting the body’s humoral immune response to altered self-proteins, such as mutated, aberrantly expressed, or post-translationally modified autoantigens [20]. This immune recognition often occurs early in tumorigenesis, making autoantibodies useful not only for diagnosis and monitoring, but also for identifying druggable targets and informing novel immunotherapeutic approaches [20]. A study conducted by Montero-Calle et al. (2023), highlights the potential of using different proteoforms of the p53 and p63 proteins as early blood-based markers for CRC [19]. By analyzing how the immune system reacts to these specific protein variants in blood samples, researchers found that certain proteoforms were more commonly recognized in patients with CRC or precancerous lesions than in healthy individuals. The results showed high accuracy in distinguishing these groups, and testing with user-friendly biosensor devices confirmed their potential for clinical use. These findings suggest that p53 and p63 proteoforms could help improve early detection of CRC through a simple blood test [19]. Moreover, autoantibodies targeting p53 are frequently associated with CRC, owing to the high mutation rate of the *TP53* gene in this malignancy [20]. While these autoantibodies exhibit high specificity in advanced CRC (89–100%), their diagnostic sensitivity remains limited (8.8–46.3%). Notably, emerging evidence suggests that p53 autoantibodies can also be detected in early-stage CRC and premalignant lesions, albeit with variable sensitivity (10–45.2%). Furthermore, autoantibodies against other p53 family proteoforms, such as p73, ΔNp73α, and ΔNp73β, have shown enhanced diagnostic performance [20]. These proteoforms may become immunogenic due to their dysregulation or mutation in CRC. Recent studies report that autoantibodies against these variants achieve sensitivities above 49%, specificities exceeding 86%, and strong discriminatory power for early detection (AUC > 65%), including in premalignant conditions [20].

LBs offer a promising platform for the non-invasive detection of PTMs of proteins, enabling dynamic monitoring of disease-related signaling alterations and treatment responses in real time. Phosphorylation, a key protein PTM, often becomes dysregulated in cancer, leading to abnormal signaling and therapy resistance. To address this, Ahmed et al. (2023) developed a multiplexed phosphoprotein analyzer chip (MPAC) capable of rapid and sensitive detection of phosphorylated proteins in lung cancer [132]. Using lung cancer cell lines and patient-derived EVs, the chip profiled major pathways (MAPK and PI3K/AKT/mTOR) and detected treatment responses, including to kinase inhibitors and immunotherapy [132]. A phosphorylation heat map from plasma samples distinguished cancer from noncancer individuals and revealed proteins associated with therapy response. This tool shows strong potential for personalized cancer treatment through dynamic phosphoproteomic monitoring.

**Figure 2:**
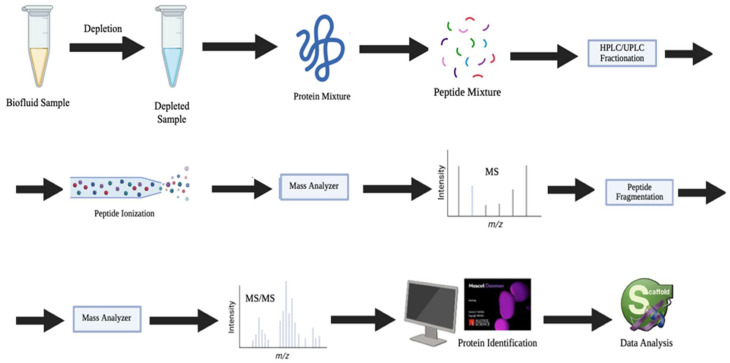
Proteomic workflow used for protein biomarker detection in liquid biopsies. HPLC/UPLC—high-performance liquid chromatography/ultra-performance liquid chromatography; MS—mass spectrometry; MS/MS—tandem mass spectrometry; *m/z*—mass-to-charge ratio.

The proteomics-based workflow for the detection of protein biomarkers in LB (Figure 2) relies widely on high-resolution liquid chromatography-tandem mass spectrometry (LC-MS/MS), a powerful platform for both discovery and targeted proteomics [133,134].

This process typically begins with the collection of biofluidsamples such as plasma, serum, urine, tear biofilm, breast milk, NAF, or saliva, followed by protein extraction and enzymatic digestion—commonly using trypsin—to generate peptide mixtures. These peptides are then separated via liquid chromatography (LC) and analyzed by tandem mass spectrometry (MS/MS), enabling precise identification and quantification of proteins. LC-MS/MS offers high sensitivity and specificity, allowing for the comprehensive profiling of the proteome, including low-abundance proteins and critical PTMs of proteins that may serve as cancer biomarkers [135]. Quantification can be performed using either label-free approaches or chemical labeling techniques such as isobaric tags for relative and absolute quantitation (iTRAQ, TMT), which enhance throughput and enable comparative analyses across multiple samples. This workflow is particularly well-suited for non-invasive liquid biopsies, as it facilitates the identification of disease-associated protein signatures that differentiate between healthy and pathological states. Moreover, LC-MS/MS supports both untargeted (discovery-phase) and targeted (validation-phase) proteomics, making it instrumental in biomarker discovery, early cancer detection, real-time monitoring of disease progression, and assessment of therapeutic response. By delivering deep proteome coverage with robust analytical performance, LC-MS/MS-based proteomics is paving the way for the development of clinically actionable biomarker panels that can significantly enhance the precision and personalization of cancer diagnostics and monitoring. 

Due to the high complexity and variability of the human proteome, there is a growing demand for innovative technologies that enable accurate and efficient biomarker discovery [47]. Recent advances in MS instruments, such as the Q Extractive HF, Orbitrap Exploris 480, timsTOF Pro 2/HT, and ZenoTOF 7600—have dramatically improved scan speed, resolution, and dynamic range, enhancing detection and quantification of low-abundance proteins [136]. 

A major advancement is this field is the integration of ion mobility technologies, such as high-field asymmetric waveform ion mobility spectrometry (FAIMS) and trapped ion mobility spectrometry (TIMS). These methods provide additional gas-phase separation, reduce background noise and increase proteome coverage, especially in complex clinical samples like FFPE tissues and low plasma volumes [137]. This improvement supports more comprehensive profiling of the proteome and proteoforms, including dysregulated species with biomarker potential. Using timsTOF-based four-dimensional label-free proteomics, Lu et al. (2025) successfully enhanced the detection of low-abundance proteins in patient-derived EVs [138]. They found that EVs from metastatic nasopharyngeal carcinoma (NPC) patients were significantly enriched compared to those from locoregional NPC and healthy individuals, and were functionally involved in processes like cell motility, metabolism, and angiogenesis [138]. 

Furthermore, the parallel reaction monitoring-parallel accumulation-serial fragmentation (prm-PASEF) method on the Bruker timsTOF Pro mass spectrometer enables highly sensitive, multiplexed, and absolute quantification of plasma proteins [139]. By integrating ion mobility, this technique improves depth and speed of analysis, quantifies proteins across a wide dynamic range, and shows excellent concordance with traditional multiple reaction monitoring (MRM), making it particularly suitable for clinical biomarker validation [139]. These technological advances in MS and ion mobility significantly enhance biomarker discovery by enabling deeper, faster, and more sensitive proteome analysis, especially from complex samples. They hold strong potential for advancing early cancer detection and precision medicine through LB applications.

The main sources for LB include a variety of bodily fluid such as blood/plasma/serum, urine, saliva or oral fluid, human breast milk, nipple aspirate fluid, pleural fluid, and aqueous humor. The following sections examine each of these liquid biopsy sources, outlining their specific advantages, biomarker content, and relevance to cancer diagnostics.

- Blood/Serum/Plasma

A recently published editorial written by Bradley and Watson (2025) showed that blood tests was the most frequently performed category in patients who were later diagnosed with cancer, 44% of patients receiving such a test [140]. PCa is the most common urological cancer in men worldwide, substantially contributing to morbidity and mortality [106,108,141]. For PCa diagnosis, the serum PSA, an important biomarker that correlates with the risk of PCa, and digital rectal examination (DRE) are the most commonly used methods, followed by biopsy of the prostate gland, considered the gold standard diagnostic technique for PCa detection [107,142]. In primary care for PCa detection in England, the PSA level obtained after asymptomatic PSA testing is useful in diagnosing one in five patients with PCa [89]. However, PSA is unable to distinguish between indolent and aggressive PCa [29]. Merriel et al. (2022) showed that PSA is highly sensitive but poorly specific for PCa detection on symptomatic patients [28]. The DRE sensitivity performed by primary care clinicians during a study conducted by Naji et al. (2018) was 51%, with 59% specificity [141]. Serum PSA is an appropriate example of serum biomarker largely used as a screening test in the diagnosis and prognostication of PCa that is organ –but not cancer-specific, its level being also elevated or extremely elevated in non-cancerous prostatic conditions, including urinary tract infection, prostatitis and benign prostatic hyperplasia (BPH) [31,32]. Streicher et al. (2019) revised the sensitivities reported for serum PSA in different studies, reporting a sensitivity of 32% for PCa detection, using a threshold value of 3.1 ng/mL, and 21% sensitivity for 4 ng/mL, leading to overdiagnosis and overtreatment of latent cancer without clinical evidence, correlating to a relatively high rate of unnecessary biopsies [142]. 

Furthermore, it is important to note that the level of serum PSA can be influenced by various internal and external factors. Thus, there are studies indicating a correlation between age and serum PSA levels in different populations [143,144]. Serum PSA level of obese men is lower than similarly aged lean men, due to hemodilution and hormonal changes based on an higher estradiol/testosterone ratio [145]. Moreover, Koc et al. (2013) showed that cigarette smoking affects hormone levels, so the PSA level could be higher in smokers compared with non-smokers [146]. Press et al. (2020) reported that cigarette smoking and tobacco use were associated with an increase in PSA among older African American men in Chicago, whereas marijuana use was inversely associated with PSA levels [147]. Ethnic group-based differences in PSA levels in men without PCa have been reported [148]. Thus, Black men have higher PSA levels than White or Hispanic men, resulting in higher PCa incidence and mortality in Black men than White men, while Asian men tend to have lower PCa incidence and mortality than White men, but the effects of ethnicity on PSA level are not clearly demonstrated [148,149]. 

Blood LB could be a valid alternative to tissue biopsy in PCa for diagnostic use, molecular profiling, as well as for discovery of predictive and prognostic biomarkers [17]. There is a pressing need for improved biomarkers to aid clinicians in diagnosis, prognostication, assessment of therapy response, and characterization of mechanisms of resistance [150].

- Urine

Urine is a complex biological fluid that is proven to give valuable insights into the physiological and pathological processes of the human body. As a filtrate of blood plasma, it contains a wide variety of biomolecules originating from the whole organism. Urine contains a wide range of proteins, including those secreted by the cells of kidneys or the urinary tract (70%), as well as those present in the systemic circulation at the time of filtration (30%) [151]. The use of proteins from urine samples for cancer detection and identification has gained significant attention in the past years. This strategy takes advantage of proteomic analysis using methods such as mass spectrometry (MS) to identify reliable biomarkers for various cancers, including breast (BC), bladder and PCa. Urine has some advantages and disadvantages. It can be collected in significant quantities, in a simple and non-invasive manner, without causing much discomfort to patients. This property of urine sampling makes it a preferred choice for the early-detection and long-term monitoring of cancer [152], as well as for individuals requiring frequent assessments. It has been shown multiple times that the proteome present in urine reflects local and systemic physiological changes in the body. Thus, its analysis can be used to assess a wide range of biomarkers related to cancer or other diseases, being especially promising for the investigation of hard-to-biopsy tissues [153]. On top of this, up-to-date proteomic techniques such as MS and proximity-extension analysis (PEA) have enabled the cost-effective and high-throughput analysis of the urinary human proteome. These methods have improved both the qualitative and quantitative identification of novel cancer biomarkers, making them well-suited for large scale clinical studies [154].

However, there are some drawbacks worth taking into consideration when using the analysis of urinary proteins as a tool for disease diagnosis and monitoring. It is well known that urine samples are characterized by very high intra- and inter-individual variability due to factors such as lifestyle, dietary patterns and renal function. This inconsistencies can slow the identification of new biomarkers and reduce the reliability of diagnostic tests [152,155].

Moreover, it is very likely that more diseases, if present in the same individual, can impact the same urinary biomarkers in different ways, thus hindering the result of these analyses. The low protein concentration of urine represents another disadvantage, making it difficult to identify and accurately quantify low-abundance biomarkers [156]. Techniques such as data-independent mass-spectrometry (DIA-MS) has shown promising results on overcoming this limitation [154]. Additionally, urine is known to contain high levels of urea, salts, and other interfering molecules that can hinder urine proteome analysis [152,157]. This limitation requires additional preparation steps that can make a clinical study more time-consuming and less cost-effecting. In addition, lack of standardized protocols for urine sample collection, processing and analysis is likely contributing to inconsistencies in study results, thus hindering the translation of experimental biomarkers into clinical settings [158].

A recent study by Kong et al. (2024) set out to identify novel bladder cancer biomarkers, revealing 40 significantly altered serum proteins and 17 urine proteins compared to healthy controls. Among them, ESM-1, AREG, RET, WFDC2, FGFBP1 and PVLR4 were successfully validated using singe-cell sequencing and IHC. Using LASSO regression, a 14 protein diagnostic model (11 from serum and 3 from urine) was created. This model achieved high-diagnostic accuracy, outperforming FISH in the detection of the early-stage and low-grade bladder cancer [18]. Another study investigating urinary proteins as cancer biomarkers successfully identified specific heat shock proteins (HSPs) with distinct expression patterns across various cancer types using machine learning (ML). HSP90AB1, TRAP1, FKBP4, HSPA9, and HSPB5 were identified to present unique signatures in urine that correlate with the presence of bladder, lung, and other cancers. Notably, low CCT2/HSP90AB1 levels were associated with non-cancer groups, while low HSP90AB1/TRAP1 and HSPA6/TRAP1 were associated with an increased chance of cancer presence. Moreover, this study also underscores the fact that isoforms of HSPs could help differentiate benign lung disease from lung cancer, reinforcing the diagnostic potential of HSP profiles in cancer detection and identification [159]. Several biofluids contain prostate-derived EVs and reports suggest that EVs from the bloodstream can be found in urine [160]. Urinary LB is emerging as a promising method for PCa detection [161]. Exosomes, as a novel type of liquid biopsy, have the potential to become diagnostic and prognostic biomarkers [162].

These up-to-date findings highlight the potential of urinary proteomics in cancer diagnosis. By harnessing advanced proteomic techniques and machine learning, urine-based liquid biopsies could offer a non-invasive, cost-effective, and sensitive approach to cancer detection and monitoring.

- Saliva or oral fluid

Saliva or oral fluid, is a clear and odorless biofluid secreted by the salivary glands, playing a crucial role in maintaining homeostasis within the oral cavity [163]. It is composed predominantly of water approximately (99%), alongside a diverse array of proteins (including enzymes, mucins, and immunoglobulins), other organic molecules (such as glucose, nucleic acids, lipids, and metabolic waste products) and electrolytes [163,164,165]. The term total human saliva refers to the final composition of this secretion, which also incorporates crevicular fluid- an inflammatory exudate derived from periodontal tissues, along with cellular debris, upper airway secretions, and microorganisms residing in the environment [166].

Salivary composition is subject to high intra- and inter-individual variability. Among the factors contributing to this variability, salivary flow rate appears to be the most influential [163]. Similarly to urine, saliva offers a major advantage in clinical applications due its easy, safe, and non-invasive collection process, making it especially suitable for repeated sampling required in cancer monitoring [167]. In recent years, saliva has emerged as a promising medium for detecting breast cancer (BC) [168] or oral and head and neck cancers [169] an others. For instance, a systematic review revealed that saliva, as a non-invasive biomarker, has the potential to accurately differentiate between BC patients from healthy controls with a pooled specificity and sensitivity of 72.7% and 71.7%, respectively [168]. Some of salivary constituents have been identified as potential biomarkers for these malignancies. A study by Krapfenbauer et al. (2014) identified a list of proteins detected by two-dimensional polyacrylamide gel electrophoresis (2DE-PAGE) in human saliva, including keratins, keratin subunits, enzymes and enzyme inhibitors, cytokines, immunoglobulins as well as amylase and other salivary specific glycoproteins, some of them significantly overexpressed in OSCC [170]. The molecular profile of saliva also carries valuable physiological and pathological information, particularly relevant to the head and neck region, potentially offering insights into cancer development and progression [171].

In addition to its diagnostic potential, saliva-based testing is cost-effective, making it a compelling option for large-scale screening initiatives. Furthermore, it poses minimal risk of cross-contamination and is safer for healthcare-workers [172]. However, despite these advantages, saliva-based liquid biopsies still face notable challenges. Chief among them is the lack of standardized protocols for sample collection, processing, and analysis, which undermines the reliability and reproducibility of biomarker identification [173,174]. Even if a real-time, non-invasive diagnostic tool for oral cancer were available, implementing a widespread screening program might still be inefficient due to the relatively low incidence of these cancers [172,174]. Moreover, salivary samples can be easily contaminated depending on various factors, such as the timing of food intake, oral bleeding, or enzymatic activity from either the host or oral microbiota. These variables can alter biomarker stability and interfere with diagnostic accuracy [172].

A recent systematic review by Bastías et al. (2024) evaluated 62 studies to identify promising salivary biomarkers for the early detection of oral carcinoma. All included studies reported high specificity and sensitivity of the proposed biomarkers [175]. This analysis covered over 60 biomolecules found in saliva samples from more than 5000 subjects, including patients with OSCC, oral potentially malignant disorders (OPMDs), and healthy controls. The authors highlighted TNF-α, IL-1β, IL-6, IL-8, LDH, and matrix metalloproteinase 9 (MMP-9) as particularly promising biomarkers for both OSCC and OPMDs. However, they showed that it is unlikely for a single biomarker to serve as a definitive diagnostic tool. Supporting this perspective, a study conducted by Ghallab and Shaker (2016) demonstrated that both chemerin and MMP-9 are significantly elevated in the serum and saliva of patients with OSCC [176]. Notably, the study found that salivary chemerin and MMP-9. As well as serum chemerin, were highly effective in distinguish OSCC patients from those with oral premalignant lesions (OPMLs). These biomarkers exhibited 100% sensitivity and 100% specificity, outperforming serum MMP-9 in diagnostic accuracy [176].

The authors advocate for the development of biomarker panels, which can more accurately reflect the complex physiological and pathological states associated with malignancy [175]. Future research should aim to validate these findings by developing and testing robust biomarker panels. Such efforts should include comparative analyses of multiple body fluids and assess the sensitivity and specificity of biomarker combinations for improved early cancer detection and monitoring.

- Human breast milk

Breast milk proteome analysis emerged as a promising method for assessing BC risk, as it provides access to molecular profiling for a high volume of breast tissue [13,177,178]. Recent research has focused on identifying possible biomarkers in breast milk that could help in the early detection, diagnosis and monitoring of BC. Mass spectrometry-based proteomics is one of the most accurate methods for the analysis of breast milk samples due to its ability for identification and quantification of proteins, but also of their isoforms, variants, and modified proteins [177]. Thus, one-dimensional (1D) SDS-PAGE (separation based on molecular weight as the only dimension) or two-dimensional (2D) SDS-PAGE (separation based on molecular weight and isoelectric point as the two dimensions), as well as 1D or 2D polyacrylamide gel electrophoresis (1D PAGE and 2D PAGE) coupled with nano-liquid chromatography-tandem MS (nLC-MS/MS) lead to accurately identification of dysregulated proteins that play a role in cancer progression and might be considered as putative BC biomarkers [179]. A series of studies revealed differential expression of certain proteins in the breast milk of BC-positive women vs. healthy controls, such as lactadherin, also known as milk fat globule-epidermal growth factor 8 (MFG-E8) or secreted protein containing EGF-like repeats and discoidin/F5/8 complement domains (SED1), lactoferrin (LF), gelsolin (GSN), alpha-amylase, vitronectin (VTN/VN), casein family proteins, albumin, etc. [177,179,180,181]. Lactadherin (MFG-E8) plays important roles in cell adhesion, angiogenesis promotion, and tissue regeneration, emerging as a biomarker of BC, and associated with poor prognosis and decreased survival not only in BC, but also in ovarian, colorectal, melanoma and other cancer types [182]. LF is an ubiquitous multifunctional iron-binding glycoprotein of the transferrin family, present in various biological fluids, such as milk, pancreatic secretion and bile, saliva, as well as in neutrophils [183,184]. LF, detected as downregulated in breast milk of BC women [185] could play a role in tumor promotion, especially in cancers of the female reproductive system and BC, mainly contributing to development of TNBC phenotype [184]. Keskin et al. (2022) reported that calprotectin (CP) and LF levels increased significantly during radiotherapy in the mouth rinse samples of patients with head- and neck-cancer, but their levels decreased significantly after the treatment, suggesting the radiation-therapy-induction of inflammatory environment in the oral cavity [186]. GSN, a calcium-regulated actin-binding protein, has the ability to be a prognosis, diagnostic, and immune biomarker in pan-cancer, mediating cytoskeletal remodeling and regulating the epithelial-to-mesenchymal (EMT) transition [187]. Evidence suggests that GSN had a good diagnostic value in serum of BC patients [187]. Alpha-amylase, upregulated in breast milk of BC women [185], was also found as significantly overexpressed in saliva of patients with oral cancer [188], and emphasized inhibitory effects on cancer cell proliferation and glucose uptake in human neuroblastoma cell lines [189]. VTN, a multifunctional adhesive glycoprotein involved in cell growth, cell adhesion, angiogenesis, ECM degradation and metastasis, was found as upregulated in breast milk of BC patients [185], but it was assessed as overexpressed in patients with metastatic undifferentiating neuroblastoma [190]. Although these putative biomarkers show promise in detecting BC, they lack the specificity to be exclusive to a single cancer type. They have been dysregulated in the development and progression of multiple malignancies, suggesting that they could be part of a pan-cancer biomarker profile. Evidence suggests that the proteomic profile of breast milk could complement other screening methods, especially for young women with dense breast tissue.

- Nipple aspirate fluid (NAF)

NAF is a natural secretion produced by the luminal epithelial cells that lines breast ducts, which can be non-invasively acquired via nipple by aspiration using a suction device, resulting in a liquid biopsy from both breasts. Thus, NAF may recapitulate the characteristics of the microenvironment of breast tumor [191]. Proteomic patterns of NAF can be obtained by use of surface enhanced laser desorption and ionization time of flight (SELDI-ToF) MS that is rapid and reproducible, identifying the differentially expressed proteins (DEPs) from BC patients compared to healthy controls, including those with abnormal mammogram but biopsy normal [192]. Thus, NAF is also a promising source of biomarkers for BC detection, emerging as an additional tool next to imaging or as a replacement tool for when application of imaging methods is not possible [193]. Many tumor biomarkers have been investigated in NAF, such as CEA and PSA [193]. As a future perspective, NAF biomarker profiling could be used for routine breast health monitoring by home-based liquid biopsy collection kits [30].

- Pleural fluid (PF)

PF can be considered as a successful source of liquid biopsy and is an useful opportunity especially in cases where tissue biopsy is not available [194]. Thus, malignant pleural effusion (MPE) arises during progression of many tumors, such as lung, breast and ovary cancer [194,195]. MPF are richer in tumor-derived products that can be clinically exploited than plasma [194].

- Tear fluid

Tears are a clear extracellular fluid secreted by the lacrimal glands, containing a diverse array of mRNA, lipids, and relatively high concentrations of proteins/peptides, including enzymes and cytokines, that could be considered as putative cancer biomarkers [196]. Thus, tears reflect systemic and local physiological changes, offering clinically relevant information from various bodily tissues [197]. Most proteins identified in plasma were found in tear samples, suggesting that tear fluid may serve as a surrogate for blood in certain diagnostic contexts [198]. A study by Daily et al. (2022) demonstrated the utility of tears as a non-invasive source for the early detection of breast cancer (BC), utilizing LC-MS/MS for protein profiling, followed by ELISA for biomarker validation [197]. These findings support the potential role of tear-based testing as a complementary tool to conventional screening methods, such as mammography [199]. Kaufmann et al. (2022) showed that protein profile in tears of patients with CRC could have a real potential as a non-invasive screening test, compared with invasive colonoscopy or other screening options with low sensitivity as the fecal immunochemical test [200]. 

In addition to proteomic analysis, human tears have also been explored as a source for studying PTMs, which may offer further insights into disease processes at the molecular level [201]. The non-invasive nature of tear collection, combined with minimal sample processing requirements and short processing times, makes tears a particularly attractive biofluid for diagnostic applications [198]. However, before tear-based testing can be adopted in clinical practice on a large scale, standardization of procedures related to sample collection, storage, and protein extraction, similar to those established for blood, is essential [198].

- Aqueous humor (AH)

In addition to commonly used biofluids, AH is increasingly recognized as a valuable source for proteomic analysis, particularly in ocular cancers like uveal melanoma (UM) and retinoblastoma (Rb), where it can provide insights into tumor biology and support minimally invasive diagnostic strategies. AH analysis in eyes affected by UM revealed significantly elevated levels of inflammatory, angiogenic, and immune-related biomarkers, such as IL-6, IL-8, CCL5/RANTES, EGF, basic fibroblast growth factor (bFGF), macrophage migration inhibitory factor (MIF), and MCP, compared to controls [202]. A borderline increase in VEGF was also observed. Notably, IL-8 levels correlated with tumor thickness, and IL-6 with serous retinal detachment, suggesting a link between biomarker concentration and tumor characteristics. These findings highlight the potential of AH proteomics to characterize the UM tumor microenvironment and contribute to improved understanding and monitoring of the disease [202]. Rb, the most common intraocular cancer in children, traditionally cannot be biopsied due to risk of tumor spread. However, recent advances in treatment now permit safe extraction of AH, offering a novel opportunity to use AH as a liquid biopsy [203]. AH biomarkers may now play a clinical role in diagnosis, prognosis, and disease monitoring during therapy. This shift marks a potential breakthrough in non-invasive Rb management [203].

### 3.2. Near-Clinical or Translational Tools

In recent years, a growing number of innovative cancer detection tools have moved beyond the proof-of-concept stage and are now being rigorously evaluated in clinical research settings. While not yet fully established in routine clinical practice, they are increasingly integrated into clinical trials and translational studies, reflecting their potential to complement or even transform existing diagnostic workflows. Their continued development and validation mark a critical step toward more precise, non-invasive, and real-time cancer care.

- Pan-cancer analysis and multi-omics approaches

Pan-cancer analysis represents a transformative strategy in oncology, enabling the identification of molecular patterns shared across different tumor types. Pan-cancer analysis, in conjunction with multi-omics strategies, facilitates cancer diagnosis by identifying patterns common to multiple cancer types, thereby leading to personalized and effective therapies [204]. For example, Sharma et al. (2024) showed that comprehensive multi-omics analysis of BC integrates proteomics, transcriptomics, and metabolomics data obtained from tumor tissues, revealing distinct long-term prognostic subtypes [205].

The Clinical Proteomic Tumor Analysis Consortium (CPTAC) has generated extensive proteomic datasets by analyzing tumor samples, previously studied by The Cancer Genome Atlas (TCGA), using advanced MS [206]. These datasets support integrative proteo-genomic research by linking protein and genomic data, particularly in colon, breast, and ovarian cancers. To ensure consistency across analyses, CPTAC developed the Common Data Analysis Platform (CDAP), which standardizes data processing from raw spectra to peptide and gene-level outputs [206]. The platform performs peak extraction, database searching, and false-discovery filtering, while also offering phosphosite localization via PhosphoRS program. Outputs are provided in accessible formats, enabling cross-sample and cross-cancer comparisons [206]. 

Using reverse-phase protein arrays (RPPA) as a valuable tool in functional proteomics for analyzing signaling pathways involved in cancer, researchers have profiled approximately 8000 samples from 32 cancer types within The Cancer Genome Atlas (TCGA), leading to the development of The Cancer Proteome Atlas (TCPA), an open-access resource [207]. To enhance its usability, a new “TCGA Pan-Cancer Analysis” module was introduced, allowing integrated, protein-focused analyses across cancers. This includes evaluating relationships between protein expression and genetic alterations, epigenetic changes, mRNA, and miRNAs, as well as building protein co-expression networks and identifying clinically relevant biomarkers [207].

Additionally, protein PTMs are critical regulators of cellular signaling in both healthy and cancerous cells. Using high-throughput MS, researchers analyzed PTM profiles in over 1100 patients across 11 cancer types through CPTAC [208]. The study uncovered shared patterns of protein acetylation and phosphorylation linked to key cancer pathways, such as DNA repair, immune response, and histone regulation. It also revealed interactions between PTMs that influence kinase activity. These findings deepen the understanding of cancer biology and may offer new therapeutic targets [208].

Such integrative approaches enable the development of precision medicine strategies, where the therapy can be tailored to the molecular profile of individual patients, rather than relying solely on conventional clinical parameters. Furthermore, pan-cancer analysis holds the potential to uncover shared bio-pathological pathways and biomarkers across different cancer types, which could lead to the development of broad-spectrum therapies.

- Artificial intelligence (AI) and machine learning (ML)

Standard AI methods for histopathology analyses have revolutionized cancer diagnosis by optimizing specialized models for each diagnostic task, aiding in both cancer detection and prognosis [209]. AI and ML technologies are increasingly employed to analyze medical imaging, histopathology slides, and panels of tumor biomarkers, while also incorporating patient history. This integration allows AI and ML to identify diagnostic and prognostic patterns that enhance the accuracy of conventional diagnostic methods. AI and ML are particularly effective in reducing human error, enabling faster, more consistent, and holistic interpretation of large and complex datasets. As large-scale datasets continue to be generated across different studies, these innovative technologies play a crucial role in managing the vast amounts of data, something conventional statistical methods often struggle with. This capability is especially important in the discovery of complex biomolecular relationship, where ML and AI can uncover patterns that might not be apparent through manual analysis [210]. 

Moreover, the integration of AI and ML into multi-omics approaches holds significant potential for improving early cancer detection and monitoring. By combining multiple biomarkers from different bodily fluids, AI-powered algorithms can enhance the sensitivity and specificity of cancer screening, enabling the cancer detection earlier, more treatable stages [211]. 

The versatility of AI and ML algorithms (MLAs) is exemplified by their broad applications in oncology. Various algorithms, such as neural networks, logistic regression, random forest, and support vector machine, have demonstrated promising results in cancer diagnosis, medical imaging analysis, personalized treatment, and even drug discovery [212,213]. These ML-based models have the ability to process and learn from vast, multi-dimensional data, facilitating the assignment of novel biomarkers, optimization of treatment strategies, and prediction of patient outcomes. As AI and ML continue to evolve, their integration into clinical workflows could pave the way for more precise, efficient, and patient-specific care. 

By analyzing large datasets, ML and AI algorithms can identify molecular signatures that distinguish cancer patients from healthy controls, as well as detect biomarkers that are crucial for accurate cancer diagnosis [214,215,216]. For example, Qureshi et al. (2024) explored the use of AI and ML in profiling pancreatic ductal adenocarcinoma (PDAC) and discovering biomarkers, focusing on the connection between distinct proteomic and metabolomics urine biomarkers of PDAC and their diagnostic value [215]. Their study found that the XGBoost classifier outperformed conventional statistical techniques, achieving 89% accuracy and 91% sensitivity in diagnosing PDAC [215]. ML has been employed to identify novel diagnostic biomarkers for non-small cell lung cancer (NSCLC) and to identify new therapeutic targets for this type of cancer [216]. 

Taking it a step further, MLAs trained on panels of biomarkers specific to certain cancers can detect subtle abnormalities in biomolecule expression patterns, even at early stages, making early diagnosis more feasible [217]. Moreover, ML-based models have the potential to predict an individual’s likelihood of developing a specific cancer, underscoring the importance of ML as a powerful tool in cancer diagnostics [217]. 

### 3.3. Advanced Proteomic Methods with Growing Translational Promise

- Protein isoforms as precision biomarkers

Distinct protein isoforms, often arising from alternative splicing of RNA and specific PTMs of proteins, can serve as highly specific biomarkers for cancer detection and chemotherapy resistance. They enable the differentiation between tumor subtypes or stages and support more personalized therapeutic strategies [12]. Ligand binding assays remain the common methods for detecting proteoforms in clinical laboratories; however, these tests can miss unexpected modifications, making MS essential for accurate analysis of proteoforms in biological samples [12]. 

For example, p95 HER2, a truncated isoform of HER2 found in about 30% of HER2-positive BCs, is associated with more aggressive disease and resistance to the monoclonal antibody trastuzumab [218,219]. Lacking the extracellular domain, p95HER2 escapes antibody binding while maintaining oncogenic activity, making it a potential biomarker for poor prognosis and treatment response [218]. CD44, a cell surface glycoprotein, is overexpressed in most epithelial tumors and is a key marker for cancer stem cells. Specific CD44 variant isoforms play crucial roles in tumor growth, metastasis, and chemotherapy resistance by activating oncogenic pathways [220]. Targeting these variants shows promise for enhancing both cancer diagnosis and therapy. In pancreatic ductal carcinoma (PDAC), aberrant isoforms of CA125/MUC16 were associated with aggressive tumor subtypes and are implicated in disease progression and metastasis [221]. These findings highlight the clinical relevance of MUC16 isoforms as both biomarkers of disease aggressiveness and therapeutic targets in PDAC.

In conclusion, the characterization of distinct protein isoforms has become increasingly important for improving cancer diagnosis, predicting treatment response, and guiding personalized therapies. Advances in detection methods, particularly mass spectrometry, have enhanced the ability to identify clinically relevant isoforms, which are associated with tumor aggressiveness and therapeutic resistance. Continued research and clinical validation of these isoform-based biomarkers hold promise for advancing precision oncology and minimizing unnecessary interventions.

- Mass spectrometry imaging (MSI)

MSI is an advanced, label-free technique that enables the spatial mapping and quantification of molecules directly within tumor tissue samples, providing detailed molecular information without the need for labels or tags [222]. Moreover, MSI has emerged as a powerful technique for the spatially resolved detection of proteoforms, including clinically relevant isoforms, directly within tissue microenvironment (TME) [223]. A recent multiplexed workflow called ”AutoPiMS”, a semi-automated top-down tandem mass spectrometry (MS^2^) approach, demonstrates the feasibility of high-throughput proteoform assignment at single-ion sensitivity [223]. Applied to human OC tissue, AutoPiMS enabled identification of 73 proteoforms up to 54 kDa at a rate of less than one minute per proteoform [223]. MSI integration revealed over 300 differential proteoforms between tumor and stroma, highlighting intratumoral heterogeneity. Fourteen, including methylated cysteine-rich protein 1 (CRIP1) isoforms, were spatially validated at 20µm resolution [223]. These findings highlight how spatially resolved proteoform analysis can reveal cancer-specific molecular signatures and support the development of tissue-contextualized biomarkers, offering new opportunities for precision oncology and isoform-driven diagnostics.

MSI offers several advantages over conventional diagnostic methods, such as histopathology or immunohistochemistry (IHC). It provides a much comprehensive and nuanced view of tumor characteristics, allowing clinicians to assess not only the presence of specific tumor antigens but also their precise localization within the tissue architecture. This ability is crucial in understanding the molecular complexity of cancer, which influence tumor behavior, metastasis, and treatment responses.

- Single-cell proteomic analysis

Mass spectrometry and microscopy-based single-cell proteomic profiling are designed to assess intratumoral heterogeneity. These innovative approaches are expected to provide novel prognostic biomarkers and facilitate the development of personalized treatments, thus advancing both cancer detection and therapy [224,225]. 

### 3.4. Innovative, Emerging Clinical Tools

- MasSpec Pen technology

To overcome histology-related limitations, the innovative MasSpec Pen technology leverages a small volume of water droplets in contact with the tissue surface for molecular extraction, followed by mass spectrometry analysis for molecular profiling of diagnostic proteins, lipids, and metabolites. This technology provide a rapid, accurate, and non-destructive diagnostic approach for human cancer tissues or tumor margin assessment during surgery, or ex vivo, in various cancer types, including pancreatic cancer (PC) [226], OC [227], BC [228] as well as lung and thyroid cancers [63]. Although MasSpec Pen is not strictly a liquid biopsy, it shares several similarities with LB techniques due to its ability to analyze a variety of biomarkers in tissue samples quickly and non-invasively. The MasSpec Pen is an automated, biocompatible, and handheld MS device that generates mass spectra leading to the identification of potential cancer biomarkers. It allows for cancer prediction with high sensitivity (96.4%), specificity (96.2%), and overall accuracy (96.3%), in addition to predicting different subtypes of lung cancer and differentiating benign from malignant thyroid tumors [63]. 

In case of PC, the MasSpec Pen demonstrated a concordance with histopathological results of 91.5%, with a sensitivity of 95.5% and specificity of 89.7% for differentiating normal pancreas from cancer [226]. For distinguishing bile duct from pancreatic cancer, MasSpec Pen achieved an overall accuracy of 95%, with a sensitivity of 92% and specificity of 100% [226]. Sans et al. (20219) demonstrated that the linear ion trap mass spectrometer could actualy discriminate between high-grade serus carcinoma of the ovary and normal ovarian tissue, achieving 100% sensitivity and 100% specificity [226]. In BC, Garza et al. (2024) reported a 95.5% agreement with postoperative histopathology results [228].

- Nanotechnology in cancer molecular diagnosis

Nanotechnology plays a crucial role in cancer molecular diagnosis by enabling the development of nanomaterials that can selectively recognize tumor-associated protein biomarkers, along with other molecules, and facilitate their visualization [11]. For example, liganded nanoclusters, as nonlinear optical contrast agents, can be used for molecular diagnostics in cancer, enhancing sensitivity and specificity of detection [11]. Nano-machines, as nanoscale mechanical devices, are able to detect, capture, and identify cancer biomarkers, or function as targeted drug delivery systems (DDSs). These devices have wide-ranging applications in cancer diagnosis and imaging, drug delivery, theranostics, and nano-surgery [229]. 

### 3.5. Future Point-of-Care (PoC) and Multiplexing Tools

The development of PoC diagnostics has emerged from advances in nanotechnology and nanomaterials. These have enabled for non-invasive, faster, easier, and more cost-effective testing, providing in-site measurements with high sensitivity and selectivity, thanks to progress in biosensor-based technologies driven by innovative nanomaterials, like metal nanoparticles, carbon nanotubes, and graphene, able to enhance sensor performance for cancer biomarker detection at ultra-low concentrations [230,231,232]. Today, nanotechnologies facilitate the real-time detection of tumor biomarkers, offering great potential for early cancer diagnosis, personalized treatment strategies, and effective cancer monitoring. As the design and functionality of nanomaterials continue to evolve, the accuracy, sensitivity, specificity, and versatility of various biosensors, such as optical nanomaterials, are expected to enhance the quality of life for cancer patients [233]. 

Recent advances in precision medicine rely on innovative technologies that are affordable, simple, and versatile enough to detect multiple molecular biomarkers in real time from various biofluids [234]. Electrochemical bioplatforms detect cancer-related biomarkers by converting biological interactions (e.g., with DNA, RNA, proteins, or metabolites) into electrical signals [232]. They offer rapid results, minimal sample preparation, and high sensitivity, enabling early-stage cancer detection. 

Electrochemical biosensor technologies became promising tools for integrating multi-omics data to support advanced diagnostics, therapy, and personalized oncology. Electrochemical biosensors have shown significant promise in the multiplex detection of BC biomarkers, surpassing traditional clinical methods like ELISA, FISH, and PCR in sensitivity [235]. These multiplexed electrochemical sensing platforms enable simultaneous detection of proteins (e.g., HER2, MUC1, and CA 15-3), miRNAs (e.g., miR-155, miR-21, and miR-16), exosomes, and cytokines (e.g., RANKL and TNF) at ultra-low concentrations, often outperforming conventional diagnostic thresholds [235]. For instance, miRNA biomarkers can be detected at femtomolar levels, while proteins such as EGFR and VEGF are measurable at picogram levels—well below ELISA detection limits. Despite their analytical superiority, clinical adoption remains limited, raising key questions about sensor integration, miniaturization, preferred bioreceptors, and regulatory hurdles. This highlights the need for continued research to translate these innovations into PoC tools for early and multiplexed BC diagnostics [235]. Recently, Dosnon et al. (2025) proposed a wearable in-pad diagnostic named MenstruAI, a microfluidic diagnostic platform integrated into menstrual hygiene pads for non-invasive, pain-free monitoring of menstruation blood-based biomarkers [236]. It enables naked-eye or smartphone-assisted detection of several biomarkers such as C-reactive protein (CRP), carcinoembryonic antigen (CEA), and cancer antigen 125 (CA-125), showing strong correlation with venous blood results [236]. This affordable and accessible technology offers a promising tool for early disease detection and improved women’s health monitoring, potentially democratizing healthcare and increasing diagnostic equity.

The main challenges for clinical implementation of biosensors include: improving selectivity in complex biological environments, miniaturizing devices for potential implantable applications, integrating data analytics and machine learning for accurate interpretation, addressing biomarker variability and validation, navigating regulatory pathways and ensuring safety, and overcoming toxicity concerns and commercial scalability [231]. Addressing these challenges requires strong interdisciplinary collaboration and innovation across research, clinical, and regulatory sectors.

## 4. Biomarkers: Challenges and Limits

While LB represents a transformative approach in oncology, its clinical utility is often limited by the non-specific nature of certain biomarkers. Molecules such as CP, although elevated in various malignancies, are also significantly expressed in a range of non-cancerous inflammatory and infectious conditions. This overlap can lead to false-positive interpretations and complicate the differentiation between cancer-related and benign processes. The reliance on non-specific biomarkers, particularly when used alone, underscores the pressing need for more refined panels that combine sensitivity with specificity, as well as the importance of interpreting LB results within a broader clinical and diagnostic context [197].

As members of the S100/calgranulins protein family, S100A8 and S100A9 play crucial roles in inflammation and have been implicated in both pro-tumorigenic and anti-tumorigenic processes, depending on the cancer type [237]. CP (also referred to as S100A8/A9), a well-established biomarker of inflammation, is a heterodimeric cytosolic protein predominantly found in myeloid cells. This calcium- and a zinc-binding protein consists of S100A8 and S100A9 subunits and play a crucial role in several cellular processed, including cell cycle progression, cell survival, apoptosis, proliferation, differentiation, and migration [238]. It has been suggested that the extracellular form of CP exerts pro-inflammatory and antimicrobial effects, whereas the intracytoplasmic S100A8/A9 complex is primarily involved in cellular development, maintenance, and survival [239]. Furthermore, the extracellular form of CP modulates cellular oxidative stress and promotes inflammation-related cancer progression through the activation of key signaling pathways such as STAT3, NF-kB, and ERK-MAPK [240]. However, due to its involvement in a wide range of inflammatory processes, CP upregulation can be detected in various disease, making its specificity relatively low [241]. 

In a comprehensive pan-cancer analysis by Wu et al. (2022), the expression of S100A8 was found to be significantly elevated in tumor tissues [242]. Overexpression of S100A8 has been demonstrated by immunohistochemistry (IHC) in tumor tissue from various cancer types, including BC [243,244], PCa [245,246], and bladder cancer [246,247]. In OSCC, increased S100A8 expression was detected using LC-MS/MS and IF [248]. Additionally, in pancreatic ductal adenocarcinoma (PDAC), overexpression was confirmed through two-dimensional gel electrophoresis, MALDI-ToF-MS/MS, Western blot (WB), and IHC analysis [249]. 

A study using IHC to analyze tissue samples from bladder cancer, prostate, renal cancer, and healthy controls, CP expression was significantly elevated in bladder cancer biopsies compared to the other tumor types and healthy controls [246]. The study also revealed that urothelial cancers are associated with elevated levels of CP in urine, quantified by enzyme-linked immunosorbent assay (ELISA), further suggesting CP as a promising biomarker for the non-invasive detection of bladder cancer [246]. Using tear fluid as a non-invasive source for early BC detection, Daily et al. (2022) identified a panel of differentially expressed proteins, among which S100A8 and S100A9 were proposed as potential biomarkers due to their elevated expression in BC patients [197]. This study employed LC-MS/MS for initial protein identification, followed by ELISA validation, thereby providing strong support for the potential use of the tear proteome in BC screening and diagnosis [197]. 

CP has the potential to serve as diagnostic, prognostic or metastatic biomarker in several types of cancer, including those related to obstetrics and gynecology [250]. Botana-Rial et al. (2020) confirmed the value of CP as a new diagnostic biomarker for pleural effusion (PE), demonstrating with good accuracy that CP levels in benign PE (BPE) samples were higher than those in malignant pleural effusion (MPE), often associated with ovarian or BC [195]. 

In their study, Zhang et al. (2023) used IHC detection to show that S100A8/A9 levels were elevated in BC compared to normal tissue, with particularly high expression linked to poor differentiation, loss of hormone receptors, and HER2 positivity. Elevated S100A8/A9 levels were predictive of a worse prognosis for BC patients [243]. 

However, Baydar et al. (2023) found no significant difference in serum CP levels, measured by ELISA, between patients with BC requesting neo-adjuvant treatment and control groups. Interestingly, they did find the serum CP levels were associated with Ki67 level [238]. Additionally, fecal CP levels were higher in patients with early-stage BC undergoing chemotherapy, likely due to colonic- and systemic-induced inflammation and gut dysbiosis [251]. Yasar et al. (2017) found that serum S100A8 and S100A9 did not differ significantly between controls and bladder cancer patients. However, both serum and urine CP levels were significantly elevated in cancer patients compared to controls [252]. Finally, Sahin et al. (2019) reported that CP expression in tumor tissue, measured in proportion to total tissue protein level, and its urinary levels, measured by ELISA, were significantly higher in patients with high grade, muscle-invasive primary bladder cancer [253]. 

In the previously cited study by Botana-Rial et al. (2020), CP levels in pleural fluid were assessed to differentiate between benign and malignant pleural effusion [195]. The study found a sensitivity of 96% and specificity of 60% for a cut-off value of ≤6233.2 ng/mL [195]. A meta-analysis by Ye et al. (2018) reported that fecal CP had a pooled sensitivity of 83% and specificity of 61% for CRC detection [254]. A strong correlation was observed between procalcitonin (PCT), the prohormone of calcitonin (CT), and CT levels in patients with medullary thyroid carcinoma (MTC), PCT emerging as a complementary MTC tumor biomarker [255]. While fecal CP demonstrates promise as a biomarker for certain gastrointestinal cancers, its sensitivity and specificity vary across studies and populations. Factors such as disease stage, assay methods, and patient’s history can influence these values. CP’s role in LB for cancer detection remains under investigation. 

Although the individual plasma biomarkers had some ability to predict cancer, the combination panel of more biomarkers have good predictive value for cancer. For example, a study by Sun et al. (2020) showed that CEA, osteopontin (OPN), and Dickkopf 1 (DKK1) protein may be involved in occurrence, progression, and migration of non-small cell lung cancer (NSCLC) [256]. The study revealed that the serum CEA sensitivity and specificity in NSCLC detection were 86.25% and 70%, respectively; the sensitivity and specificity of serum OPN were 71.61% and 91,25%, respectively; the sensitivity and specificity of serum DKK1 were 92.5% and 65%, respectively; the sensitivity and specificity of further combination of serum CEA and OPN were 87.5% and 86.67%, respectively; the sensitivity and specificity of the combination of serum CEA and DKK1 in the diagnosis of NSCLC were 92.5% and 76.67%, respectively [256]. These authors concluded that these three methods can be used as biological markers for the diagnosis of NSCLC [256]. A panel called the four-protein biomarker panel (4MP), including pro-SFPB, CA125, CYFRA21-1, and CEA, has been validated for assessment of lung cancer risk [257]. This panel, has a sensitivity of 63% and a specificity of 83% [258]. Additionally, a study by Nolen et al. (2015) revealed that urine biomarkers may provide screening and diagnostic properties which exceed those reported for serum biomarkers and are necessary for clinical development [259].

## 5. Evaluation of Diagnostic Performance in Cancer Detection for Clinical Applications 

An analysis of the sensitivity and specificity values for several commonly used cancer detection methods allows for a comparative assessment, leading to several important considerations regarding the reliability, effectiveness, and clinical applicability of these diagnostic approaches (Table 3). Fine needle aspiration biopsy/cytology (FNAB/FNAC) and core needle biopsy (CBB) stand out with high sensitivity (74 or 97%) and high specificity (96% and 98%, respectively) for BC detection [67,68,69]. These methods are highly reliable in detecting BC, particularly with the highest accuracy reported for CNB. ^18^F-fluorodeoxyglucose Positron Emission Tomography/Computed Tomography (FDG-PET/CT) shows impressive sensitivity (97.4%) and specificity (91.2%), making it effective for detecting distant metastasis in BC [99]. Thus, the specificity and sensitivity of FDG-PET/CT were significantly higher than those of conventional imaging (CT, US, radiography, and skeletal scintigraphy), with 85.9% sensitivity and 67.3% specificity for detection of distant BC metastases [99]. Modern film mammography shows high sensitivity (71.2% for biennial screening) and good specificity (92.6%) for asymptomatic Japanese women aged 40-49 years [90]. Digital mamography demonstrate high sensitivity (97%) but moderate specificity (64.5%), indicating its utility in early detection but challenges in differentiating malignant from benign cases without further diagnostic procedures [87,88]. mpMRI (multi-parametric MRI), with a sensitivity of 93%, is a valuable tool for PCa detection, although its specificity is lower (48%) [142]. Concluding here, FNAB/FNAC, FDG-PET/CT, mammography, and mpMRI are considered to have high sensitivity in cancer detection. HE4 has demonstrated the highest specificity (97.87%) in diagnosis of epithelial ovarian cancer (EOC), making it reliable in distinguish malignant from benign cases, particularly in later stages [43]. Transrectal ultrasonography (TRUS)-guided biopsy and human epididymis protein has specificity as a strength in diagnostic methods. Thus, TRUS-guided biopsy shows high specificity (96%) for PCa detection and has moderate sensitivity (48%) [142].

Among the cancer detection methods characterized by moderate to low sensitivity, several examples can be highlighted. BSE, although simple and non-invasive, shows low sensitivity (20-30%) but relatively high specificity (87.4%). While it is not reliable for primary BC detection, it can still serve as a complementary technique alongside more advanced methods [83,260]. Serum-PSA testing, with sensitivity ranging from 21% to 32% depending on the threshold, is similarly limited. It is particularly unreliable for detecting PCa at lower PSA levels, as it often fails to identify early-stage disease [142]. In CRC screening, fecal CP at a CR50 µg/g cutoff offers moderate sensitivity (83%) but lower specificity (61%), making it a useful, though not definitive, screening tool [261]. Additionally, CP has demonstrated high sensitivity (96%) in distinguishing between benign and malignant pleural effusions, suggesting promising potential for non-invasive lung diagnosis [195].

Serum CEA levels used in detection of non-small cell lung cancer (NSCLC) exhibit relatively low specificity (70%) despite a sensitivity of 86.25%. This limitation is partly due to CEA elevation in various other cancer (such as breast, prostate, and gastric cancer), as well as in non-cancerous conditions like cirrhosis and COPD [256]. These findings highlight the importance of employing multi-biomarker panels to increase diagnostic accuracy and minimize false positive results. For instance, the combination of serum CEA and OPN demonstrates high sensitivity (87.5%) and improved specificity (86.67%), showing potential for more accurate detection and differentiation of lung and other cancers [256]. Similarly, pairing serum CEA with DKK1 yields an even higher combined sensitivity (92.5%) and moderate specificity (76.67%), representing a promising diagnostic approach for malignancies such as non-small cell lung cancer (NSCLC) [256]. The combination of matrix metalloproteinase 9 (MMP9) and KPYM (pyruvate kinase M1/2) exhibited 94% sensitivity and 87% specificity for detecting endometrial cancer (EC), while the combination catenin beta 1 (CTNB1), exportin 2 (XPO2), and macrophage-capping protein (CAPG) achieved 95% sensitivity and 96% specificity for the discrimination of endometrioid EC and serous EC [262]. These biomarkers were measured in the fluid fraction of uterine aspirates by liquid chromatography-parallel reaction monitoring (LC-PRM) and have been analyzed using the latest generation of targeted-MS aquisition [262].

The development of multi-protein biomarkers panels that combine high-sensitivity and high-specificity markers is crucial for enhancing diagnostic accuracy and minimizing misdiagnosis. LB-based strategies are increasingly recognized for their potential in non-invasive, real-time monitoring of cancer progression and evaluation of therapeutic response, although further research id required to improve their specificity. The integration of AI and ML in the analysis of large datasets generated from diverse detection methods may help overcome current linitations by anabling more personalized and precise diagnoses. Given the wide variability in sensitivity and specificity among existing cancer detection techniques—some offering high sensitivity at the expense of specificity, and vice versa—a multimodal, multi-biomarker approach, supported by AI and ML advancements, presents a promising path toward more accurate, reliable, and rapid cancer detection.

The integration of conventional cancer detection methods—such as imaging techniques, tissue biopsies, and serum-based markers—with proteomics-based LB offers a powerful, complementary approach to cancer diagnosis and monitoring. While conventional methods remain the cornerstone for initial detection and staging, they often lack sensitivity for early-stage disease or are invasive in nature. Proteomics-based LB, by analyzing protein signatures from blood or other body fluids, provides a minimally invasive tool capable of capturing dynamic changes in the tumor environment. When combined, these approaches can enhance diagnostic accuracy, enable earlier detection, and allow for more precise tracking of disease progression and treatment response. This synergy not only improves patient outcomes but also supports the move toward more personalized oncology care.

**Table 3:** Comparative sensitivity and specificity of different methods used in cancer detection.

Detection Methods	Sensitivity	Specificity	Clinical Applications
BSE	20% to 30%; 58,3%	87.4%	BC detection [83,260]
iBE	34.3% to 86%,	59% to 94%	BC detection [85]
CBE	40% to 69%	-	BC detection [263]
CBE (average-risk women)	-	99.4% (screening); 68.7% (diagnostic)	[264]
CBE (increased-risk women)	-	97.1% (screening) 57.1% (doagnostic)	
DRE	51%	59%	PCa detection [141]
serum PSA	32% (3.1 ng/mL); 21% (4 ng/mL)	-	PCa detection [142]
US	80.1% (overall pooled sensitivity)	88.4%	BC diagnosis [87]
US	89.2% (in low and middle-income country)	99.1%	
mammography	77–95%	-	BC detection [263]
modern film mammography alone, without CBE, among asymptomatic Japanese women (40-49 years)	47.4%	-	BC screening [90]
biennial modern film mammography, without CBE, among asymptomatic Japanese women (40-49 years)	71.7%	92.6%	
digital mammography	97%	64.5%, (89.3% accuracy)	BC detection [87,88]
FNAB/FNAC	74%/97%	96% (95% accuracy)	BC detection [67,68,69]
CNB	95%/97%	98% (96% accuracy)	BC detection [67,68,69]
conventional imaging (CT, US, radiography, skeletal scintigraphy)	85.9%	67.3%	detection of BC distant metastasis [99]
salivary tests	71.7%	72.7%	BC detection [168]
salivary chemerin and MMP-9, and serum chemerin	100%	100%	differentiating OSCC from OPMLs [176]
CEUS (polled sensitivity)	70%	74%	PCa detection [265]
grey-scale TRUS	40% to 50%	40% to 50%	
TRUS-guided biopsy	48%	96%	PCa detection [142]
mpMRI	93%	48%	
mpMRI and PI-RADS	89%	73%	PCa detection [142,266]
MRI	72%	96%	PCa staging [142]
FDG-PET/CT	97.4%	91.2%	detection of BC distant metastases [99]
latest-generation digital PET/CT	96.4%	86.4%	detection of cervical LN metastases in HNSCC [100]
HE4	76% to 88% (depending on disease stage)	97.87%	OC detection [43]
4MP (pro-SFPB, CA125, CYFRA21-1, CEA)	63%	83%	distinguishing benign from malign indeterminate pulmonary nodules [258]
serum CEA	86.25%	70%	NSCLC diagnosis [256]
serum OPN	71.61%	91.25%	
serum DKK1	92.5%	65%	
serum CEA and OPN	87.5%	86.67%	
serum CEA and DKK1	92.5%	76.67%	
MMP9 and KPYM (uterine aspirate)	94%	87%	EC detection [262]
CTNB1, XPO2, and CAPG (uterine aspirate)	95%	96%	discrimination of endometrioid EC and serous EC [262]
blood test based on EVs	71.2%	99.5%	detection of OC, PC, bllader cancer (early-stage) [16]
calprotectin (CP) (≤6233.2 ng/mL cutoff)	96%	60%	benign and malignant pleural effusion differentiation [195]
fecal CP (50 µg/g cutoff)	83%	61%	CRC detection [254]
	76.9%	88%	esofago-gastric cancer detection [261]
MasSpec Pen technology	95.5%	89.7%	differentiation between normal pancreas and PC [226]
	92%	96.2%	differentiation between bile duct and pancreatic cancer [226]
	96.4%	96.2%	prediction of cancer subtype of lung and thyroid cancer [63]

Abbreviations: BC—breast cancer; BSE—breast self-examination; CA19-9—carbohydrate antigen 19-9; CAPG—macrophage-capping protein; CBE—clinical breast examination; CEA—carcinoma embryonic antigen; CEUS—contrast-enhanced ultrasound; CNB—core needle biopsy; COPD—chronic obstructive pulmonary disease; CRC—colorectal cancer; CT—computed tomography; DKK1—Dickkopf-1; DRE—digital rectal examination; EC—endometrial cancer; EVs—extracellular vesicles; FDG-PET/CT—^18^F-fluorodeoxyglucose Positron Emission Tomography/Computed Tomography; FNAB/FNAC—fine needle aspiration biopsy/cytology; 4M—four-protein biomarker panel; GC—gastric cancer; HE4—human epididymis protein 4; HNSCC—head and neck squamous cell carcinoma; iBE—Intelligent Breast Exam; KPYM—pyruvate kinase M1/2; LN—lymph node; MMP9—matrix metalloproteinase 9; mpMRI—multi-parametric MRI; MRI—magnetic resonance imaging; NSCLC—non-small cell lung cancer; OC—ovarian cancer; OPMLs—oral premalignant lesions; OSP—osteopontin; PC—pancreatic cancer; PCa-prostate cancer; PI-RADS—Prostate Imaging Reporting and Data System; PSA—prostate-specific antigen; SCLC—small cell lung cancer; TRUS—transrectal ultrasound; US—ultrasonography; XPO2—exportin 2.

## 6. Future Perspectives

Despite significant advances in cancer diagnosis, the search for ideal methods to detect malignant tumor remains ongoing. Researchers, clinicians, and even patients, particularly from the perspective of participatory medicine, continue to seek more reliable tools and solutions [1]. Early cancer detection, with minimal discomfort, enables patients to begin personalized therapy sooner, thereby improving the likelihood of a successful outcome and reducing the physical and physiological impact of invasive procedures. 

However, all current methods used in cancer screening and diagnosis have notable limitations. Conventional approaches often suffer from reduced sensitivity, specificity, and accuracy. They are often invasive, limited to certain types of cancer, and typically detect one or few cancer biomarkers. Furthermore, they methods struggle to monitor the dynamic development of tumors or capture the full molecular landscape within tumors and cancerous cells. In addition, some conventional methods carry inherent risk of complications, are time-consuming, labor-intensive, and resources-consuming, and may be limited in accessibility due to high costs. Consequently, these limitations often lead to late cancer detection, overdiagnosis, overtreatment, unnecessary surgery, or delayed treatment decisions. In some cases, results are also subject to interpretation variability and human error.

This review examines the evolution of cancer detection methods, highlighting the transition from conventional techniques to emerging innovation such as liquid biopsy (LB) and molecular biomarkers. It emphasizes the need for enhanced accuracy, reduced invasiveness, and early cancer detection, as well as the facilitation of personalized treatment strategies.

A comprehensive overview is provided on various cutting-edge approaches, including multimodal imaging, genomic testing, and pan-cancer analysis, in conjunction with multi-omics strategies, which offer a more holistic understanding of cancer biology. The review also explores the growing role of AI and ML in improving diagnostic accuracy and predicting treatment response. Additionally, we highlighted the potential of proteome-based plasma tests and LB, including the sensitive detection of CTCs, which enable non-invasive monitoring of cancer progression and treatment efficacy. The use of extracellular vesicles (EVs) in protein-based blood tests represents another promising avenue, offering insights into tumor dynamics, molecular signatures, and intra-tumoral heterogeneity. 

Furthermore, single-cell mass spectrometry analysis of multiplex proteomes and microscopy-based functional single-cell proteomic profiling provide in-depth examination of tumor intraheterogeneity and the dysregulated pathways that drive carcinogenesis and tumor development. We also discuss the development of MSI and MasSpec Pen technologies, which can revolutionize cancer detection by offering rapid, accurate, and accessible testing outside conventional clinical settings.

The development of PoC diagnostic tool is accelerating the speed, ease, cost-effectiveness, and on-site availability in cancer testing, thanks to advances in biosensor technologies powered by innovative nanomaterials [230].

Today, nanotechnologies enable real-time detection of tumor biomarkers, paving the way for early diagnosis, personalized treatment plans, and continuous cancer monitoring. As the design and functionality of nanomaterials continuous to evolve, improvements in biosensor accuracy, sensing performance, specificity, and versatility will greatly enhance the quality of life for cancer patients. Recent progress in LB using optical nanomaterials offers the opportunity to discover novel biomarkers and deepen our understanding of cancer-related mechanisms. LB protein biomarker profiling could be integrated into routine health monitoring through home-based LB collection kits.

Together, these advancements are creating a pathway for more personalized, precise cancer detection and management. As these technologies evolve, they hold immense potential to enhance early detection, improve diagnostic accuracy, and optimize treatment strategies. Ultimately, these innovations offer hope for more effective and less invasive methods to fight against cancer.

Over next decades, LB is likely to evolve into a routine, highly precise, and universally accessible tool that redefines not only cancer detection, but also overall health monitoring. 

The future of LB may include:

- Ultra-early cancer detection, on even cellular or molecular level, identifying malignant transformation of cells and signaling pathways before they become anatomically detectable;
- Wearable or implantable biosensors continuously sampling and analyzing blood or interstitial fluids in real-time, alerting both patient and physician to molecular changes before symptoms appear;
- Fully personalized protein biomarker panels generated through ML and AI, dynamically adapting to individual genomic and proteomic profiles, lifestyle factors, and environmental exposures;
- PoC diagnostics in everyday life, including smartwatches or home health stations, enabling regular health checks without needing clinical visits;
- Integration with preventive medicine, where LB not only detects cancer but predicts risk, monitors predisposition, and guides proactive interventions years before clinical cancer manifests.

In this feature, the role of tissue biopsy may become limited to rare or complex cases, while LB becomes the foundation of longitudinal health tracking, combining convenience with unprecedented depth of biological insights. 

Going further, LB could become an inseparable part of routine life, converging to a real-time, continuous monitoring of the body molecular and cellular health, where nanobots circulate in the bloodstream and collect and transmits real-time data on cancer biomarkers, disease progression, and overall health, allowing for immediate detection of even slightest abnormalities, far before symptoms emerge. These nanobots will also be equipped to adjust dysregulated signaling pathways. Advanced LB systems will be able to detect at early stage not just cancer, but also for a wide range of diseases. Cancer will be detected on a molecular level. Consequently, human lifespan may see a significant increase sue to the continuous monitoring of biomarkers, allowing for precise management of aging and prevention of age-related diseases, including cancer. Last, but not least, medical care will be more preventive and predictive, and patients will be deeply involved in participatory medicine. Furthermore, it will be an era of preemptive medicine, that refers to an advances approach in healthcare, where the focus is on preventing cancer before its even occurs rather just treating it once it manifests.

## 7. Conclusions

Cancer detection has evolved significantly. Transitioning from conventional methods to innovative, non- or minimally invasive approaches aims to enhance early diagnosis, precision, and treatment outcomes. This review highlights current and emerging diagnostic technologies, including LB, molecular biomarkers, and AI.LB, utilizing biomarkers in bodily fluids. It offers a method for monitoring cancer progression, detecting tumors at molecular levels, and predicting treatment responses. Despite its promising potential, the clinical application of LB faces several challenges. This includes issues related to sensitivity and specificity, as low levels of tumor biomarkers in early-stage cancers can lead to false negatives, while non-cancerous conditions may produce false positives. Additionally, the heterogeneity of tumors complicates the detection of all cancer types, and the lack of standardized protocols for sample collection, processing, and analysis creates variability in results. Technological limitations in detecting low concentrations of biomarkers and the high cost of advanced equipment also pose significant barriers to widespread adoption. Furthermore, the interpretation of LB results remains complex, requiring clear clinical guidelines and established threshold levels for reliable decision-making. Ethical and privacy concerns regarding patients’ data, alongside the need for large-scale validation and clinical integration, hinder the universal application of LB. While LB holds great promise for revolutionizing cancer detection, overcoming these challenges is critical to ensuring its accuracy, accessibility, and integration into routine clinical practice.

Proteoforms, the diverse variants of proteins arising from genetic and PTMs, play a crucial role in the molecular landscape of cancer. Their specific expression patterns often reflect tumor presence, subtype, and progression more accurately than conventional biomarkers. Detecting these proteoforms, especially through minimally invasive LBs, offers a promising avenue for early cancer diagnosis when intervention can be most effective. By capturing the complexity and heterogeneity of tumors at the proteoform level, clinicians can improve diagnostic precision, tailor treatments to individual patients, and ultimately enhance clinical outcomes. Ongoing innovations in proteoform detection technologies are therefore essential to realizing the full potential of personalized oncology.

Continued advancements in technology, standardization, and bioinformatics, along with ongoing research into the clinical significance of LB biomarkers, are essential for realizing the full potential of LB in cancer care. The integration of AI and ML further enhances diagnostic accuracy by enabling rapid data analysis from diverse sources, including genomics, proteomics, and imaging. Additionally, advancements in nanotechnology, single-cell analysis, and mass spectrometry are reshaping the landscape of cancer detection, enabling high-sensitivity, real-time monitoring of biomarkers. Despite the progress, challenges such as limited specificity, high costs, and the need for multi-biomarker panels remain. The future of cancer diagnostics is poised to focus on personalized, real-time detection using wearable biosensors, lab-on-a-chip technologies, and advanced LB systems, promising enhanced early detection, reduced invasiveness, and improved patient outcomes. In the long term, these innovations may redefine cancer detection and treatment, leading to a more proactive, predictive, and patient-centered healthcare approach.

## Figures and Tables

**Table 1:** Histology vs. LB for cancer diagnosis, monitoring, and treatment.

Features	Histology	LB
Limitations	limited for certain types of cancer	potential for detection across multiple cancer types
	limited to one or few biomarkers by multiplex detection in IHC	panel/multiple biomarkers assignment
	time consuming, labor-intensive	detection of low-abundant proteins can be challenging; faster results
Invasiveness	invasive	non-invasive or minimally invasive
	multiple biopsies are not feasible due to their invasive nature	can be performed more frequently with fewer risks to patient
Accuracy	high specificity, depends on sample quality, interpretation variability and human error	high specificity and sensitivity, especially with advanced technologies
Risk to patient	infections, bleeding, pain, and prolonged recovery	minimal to no risk
Sample type	tissue sample, usually from biopsy or surgical resection	detect CTCs, DNA, EVs, proteins, metabolites from multiple tumor regions
Monitoring and fellow-up	provides a snapshot of the tumor at the time of biopsy	allows for real-time monitoring of cancer
Molecular insights	inability to monitor dynamic changes, provides limited molecular insights, focusing on histological features	dynamic insight, provides a broader range of molecular biomarkers
Early cancer detection	risk of missing early or small lesions, limitation in detection of early-stage cancer or micrometastases	allows for early detection of cancer and minimal residual disease
Detection of tumor heterogeneity	limited; may not capture the clonal and molecular diversity within the tumor	can detect molecular variations across the entire tumor mass, including mutations and PTMs of proteins
Cost	generally higher	generally lower
Treatment decision-making	delayed decision-making for treatment	accelerate decision-making for treatment

**Table 2:** Computed tomography (CT) vs. LB for cancer diagnosis, monitoring, and treatment.

Features	CT	LB
Principle	considered non-invasive, possible negative effects due to dose-cumulative effects of X-rays	non-invasive/minimally invasive
Diagnostic ability	high-resolution images for detecting tumors, distant metastasis, and anatomical changes	detects molecular biomarkers such as mutations, gene expression, and tumor-related proteins
Sensitivity	high sensitivity for detecting structural changes, particularly in large tumors	depends on the biomarker used and the cancer stage but can detect early-stage cancers
Specificity	varies among tumor types, size, location	depends on the biomarker panel
Tumor monitoring	effective for monitoring tumor size and response to treatment	monitors continuously and non-invasive the response to therapy
Costs	can be expensive, necessitates imaging equipment	less expensive than conventional imaging, the costs depends on the complexity of the test
Clinical applications	commonly use in cancer detection, staging, monitoring, and evaluating metastasis	used for early-stage cancer detection, monitoring minimal residual disease, and assessing treatment efficiency; earlier detection of metastasis at molecular level
Use in early detection	detects tumors that have reached a certain size	detects cancer even before clinical signs are present
Advantages	high spatial resolution accuracy, effective for visualizing tumor anatomy	provides molecular insights into tumors, can be done repeatedly, and is non- or minimally invasive
Disadvantages	limited in early-stage cancer detection, X-ray exposure is disputable; not detects molecular characteristics	many methods require additional validation, can have lower sensitivity for certain tumor types
